# Alterations to DNA methylation patterns induced by chemotherapy treatment are associated with negative impacts on the olfactory pathway

**DOI:** 10.1186/s13058-023-01730-4

**Published:** 2023-11-06

**Authors:** Peh Joo Ho, Alexis Jiaying Khng, Benita Kiat-Tee Tan, Geok Hoon Lim, Su-Ming Tan, Veronique Kiak Mien Tan, Ryan Shea Ying Cong Tan, Elaine Hsuen Lim, Philip Tsau-Choong Iau, Ying Jia Chew, Yi Ying Lim, Mikael Hartman, Ern Yu Tan, Jingmei Li

**Affiliations:** 1 Genome Institute of Singapore (GIS), Agency for Science, Technology and Research (A*STAR), 60 Biopolis Street, Genome, Singapore, 138672 Republic of Singapore; 2 Saw Swee Hock School of Public Health, National University of Singapore and National University Health System, Singapore, Republic of Singapore; 3 Department of Surgery, Yong Loo Lin School of Medicine, National University of Singapore and National University Health System, Singapore, Republic of Singapore; 4 Department of Breast Surgery, Singapore General Hospital, Singapore, Republic of Singapore; 5 Division of Surgery and Surgical Oncology, National Cancer Centre Singapore, Singapore, Republic of Singapore; 6 Department of General Surgery, Sengkang General Hospital, Singapore, Republic of Singapore; 7 KK Breast Department, KK Women’s and Children’s Hospital, Singapore, 229899 Republic of Singapore; 8 Division of Breast Surgery, Changi General Hospital, Singapore, Republic of Singapore; 9 Division of Medical Oncology, National Cancer Centre Singapore, Singapore, Republic of Singapore; 10 Oncology Academic Programme, Duke-NUS Medical School, Singapore, Republic of Singapore; 11 Department of Surgery, University Surgical Cluster, National University Health System, Singapore, 119228 Singapore; 12 Department of General Surgery, Ng Teng Fong General Hospital, 1 Jurong East St 21, Singapore, 609606 Republic of Singapore; 13 Department of General Surgery, Tan Tock Seng Hospital, Singapore, 308433 Republic of Singapore; 14 Lee Kong Chian School of Medicine, Nanyang Technological University, Singapore, Republic of Singapore

**Keywords:** Epigenetic modification, Breast cancer, Treatment response, DNA methylation

## Abstract

**Background:**

Exposure to cytotoxic chemotherapy treatment may alter DNA methylation (DNAm) in breast cancer patients.

**Methods:**

We performed DNAm analysis in 125 breast cancer patients with blood drawn before and after chemotherapy, using the Illumina MethylationEPIC array. DNAm changes of 588,798 individual CpGs (including 41,207 promoter regions) were evaluated using linear regression models adjusted for monocyte proportion. Gene set enrichment analyses (GSEA) were conducted to identify key Gene Ontology (GO) biological processes or Kyoto Encyclopedia of Genes and Genomes (KEGG) pathways associated with chemotherapy. Results were validated in a separate cohort of breast cancer patients who were treated (*n* = 1273) and not treated (*n* = 872) by chemotherapy (1808 blood, 337 saliva).

**Results:**

A total of 141 differentially methylated CpGs and 11 promoters were significantly associated with chemotherapy after multiple testing corrections in both the paired sample and single time point analyses. GSEA of promoter regions (pre-ranked by test statistics) identified six suppressed biological processes (*p* < 4.67e−8) related to sensory perception and detection of chemical stimuli, including smell perception (GO:0007606, GO:0007608, GO:0009593, GO:0050906, GO:0050907, and GO:0050911). The same six biological processes were significantly suppressed in the validation dataset (*p* < 9.02e−14). The KEGG pathway olfactory transduction (hsa04740) was also found to be significantly suppressed (*p*_paired-samples_ = 1.72e−9, p_single-timepoint-blood_ = 2.03e−15 and *p*_single-timepoint-saliva_ = 7.52e−56).

**Conclusion:**

The enrichment of imprinted genes within biological processes and pathways suggests a biological mechanism by which chemotherapy could affect the perception of smell.

**Supplementary Information:**

The online version contains supplementary material available at 10.1186/s13058-023-01730-4.

## Background

DNA methylation (DNAm), a form of dynamic and reversible epigenetic regulation, is highly sensitive to environmental pressures on the body [1, 2]. Hence, the cytotoxic nature of chemotherapy is expected to have a profound impact on a patient’s DNAm landscape [3, 4].

In recent years, there is an increasing number of studies looking into the mechanistic, biomarker, and therapeutic roles of epigenetic pathways in response to chemotherapy [3, 5]. Yao et al. characterized epigenetic changes in paired pre- and post-chemotherapy blood specimens from 93 breast cancer cases and found a marked impact on the leukocyte DNA methylome—4.2% of the CpG probes tested were significantly altered [3]; significant changes in the abundance of CD4 + T cells, B cells, and monocytes were observed. The authors highlighted specific CpG sites in four genes, *VMP1/MIR21*, *CORO1B*, *SDK1*, and *SUMF2*. Notably, CpG cg16936953 in *VMP1/MIR21* was also the most significant locus in an independent study by Smith et al. comparing patients treated with chemotherapy and those untreated (*n* = 61 breast cancer patients) [4]. In a study by Sigin et al. using 62 breast biopsy samples, differential methylation in 10 genes (*SLC9A3*, *C1QL2*, *DPYS*, *IRF4*, *ADCY8*, *KCNQ2*, *TERT*, *SYNDIG1*, *SKOR2,* and *GRIK1*) was reported to help discriminate response to neoadjuvant chemotherapy [6].

Using a larger cohort, this study aims to validate and extend the results obtained from previous studies that have investigated chemotherapy-induced epigenetic changes. We examined chemotherapy-induced changes in DNAm in 125 Asian breast cancer patients with paired blood specimens before and after treatment and validated the findings in a further 2145 patients treated (*n* = 1273) and not treated with chemotherapy (*n* = 872).

## Methods

### Study populations

#### Patients with paired specimens collected pre- and post-chemotherapy

Clinical and treatment data is retrieved from the Tan Tock Seng Hospital (TTSH, a tertiary hospital in Singapore) Prospective Breast Cancer Database (NHG DSRB Ref: 2019/00051). Between 2013 and 2023, 3243 patients gave consent to have their data stored in the database, of whom 2004 (61.9%) contributed blood specimens. A total of 130 female patients diagnosed between 2012 and 2021 had blood drawn before and after chemotherapy patients and were included in this study. For 30 patients who received neoadjuvant chemotherapy, the median time between blood draw to the start of treatment was 10 days. The median time from treatment end to blood draw was 47 days. For the remaining patients who received adjuvant chemotherapy, the time from blood draw to start of treatment and time from treatment end to blood draw was 40 and 448 days, respectively.

#### Single time point specimens from patients treated and not treated with chemotherapy

The Singapore Breast Cancer Cohort (SGBCC) comprises adult incident and prevalent breast cancer patients from seven participating public hospitals in Singapore [7]. These hospitals diagnose and treat ~ 76% of all breast cancer cases in Singapore. The overall participation rate of SGBCC as of 2016 was 86%, with 5,931 individuals (76%) providing bio-specimens [7]. As SGBCC is an on-going study, we included patients recruited from 2009 to 2018. Blood or saliva specimens were collected once at recruitment. Clinical data on tumour characteristics and treatment modalities were obtained through medical records. Samples were prioritized for selection to be included in the DNAm experiments based on the completeness of clinical and follow-up data.

### DNA isolation

Experimental details on DNA isolation of blood and saliva specimens have been previously published [8]. Briefly, whole blood specimens were centrifuged at 2500 rpm for 15 min at 4 °C to isolate buffy coats. Genomic DNA was extracted from buffy coats using the FlexiGene DNA Kit (Qiagen). DNA from saliva specimens collected using the Oragene kit (OG-500) was extracted using the prepIT-L2P DNA extraction kit (DNA Genotek).

### Bisulfite conversion

Treatment with sodium bisulfite was performed on 800 ng DNA (quantified by NanoDrop) in 96-well plates using the EZ DNA Methylation-Gold Kit (Zymo Research, Orange, CA, USA) following the manufacturer’s protocol. The modified DNA was then hybridized to the Infinium MethylationEPIC BeadChip (Illumina Inc., San Diego, CA, USA) for whole-genome DNAm profiling following the manufacturer’s protocol.

### Processing of DNAm data

Data import, quality control, filtering, and normalization of DNAm data (raw signal intensities in IDAT files) were performed using the R package RnBeads [9]. Global DNAm levels (total level of 5mC content in a sample relative to total cytosine content) were computed for each sample.

#### Sample exclusions

For the paired sample dataset, DNAm profiles were generated for 453 samples (130 unique subjects). Fifty-eight unique samples failed quality control based on MethylationEPIC internal quality control probes (“NEGATIVE”, *n* = 9; “SPECIFICITY”, *n* = 48) or were flagged as unreliable by the iterative Greedycut algorithm (*n* = 1) [9]. A further 142 duplicate samples were removed. Of the remaining 127 pre-treatment and 128 post-treatment samples, 125 individuals had paired samples included in this study.

For SGBCC, DNAm profiles were generated for 2,364 samples (workflow carried out in five batches due to a limitation in computational power). We excluded duplicated samples (*n* = 9) and samples that failed quality control based on MethylationEPIC internal quality control probes (“BISULFITE CONVERSION I”, *n* = 3; “NEGATIVE”, *n* = 50; “SPECIFICITY I”, *n* = 13; “STAINING”, *n* = 6) [9], flagged as unreliable by the iterative Greedycut algorithm (*n* = 5) and unknown sample type (*n* = 32). The total number of samples retained was 2,246. For downstream analysis, we further excluded three samples that had immune cell content (LUMP, leukocytes unmethylation for purity described below) scores < 0.9 and 98 with unknown date of chemotherapy (Additional file 1: Fig. S1). The final number of samples included in this study was 2,145.

#### Probe exclusions

Additional file 2: Table S1 lists the options of the executed module for probe exclusion. Probes were removed if (1) they overlapped with single nucleotide polymorphisms (SNPs), (2) their sequences were non-specific and had a high likelihood of cross-hybridization, or (3) they were considered unreliable measurements by the iterative Greedycut algorithm (detection *p*-value = 0.01) [8]. Background subtraction was performed using the methylumi package (method “enmix.oob”) [10]. Methylation beta values were then normalized using the beta-mixture quantile (BMIQ) normalization method [11]. Context-specific (CC, CAG, CAH, CTG, CTH, Other) probes and consistent probes with beta values which exhibited standard deviation lower than 0.005 were excluded in a second filtering step. Missing values were inferred by *k*-nearest neighbors’ imputation. As this is a female-only population, we included probes on chromosome X. Chromosome Y probes and promoters were removed. A summary of the probes removed by different criteria is given in Additional file 2: Table S2.

### Quantifying DNAm heterogeneity

DNAm is highly specific to different types of cells [12]. A complex combination of various cell types are found in blood (e.g. neutrophils, eosinophils, basophils, CD14+ monocytes, CD4+ T cells, CD8+ T cells, CD19+ B cells, and CD56+ natural killer cells) and saliva samples (e.g. leukocytes and epithelial cells) [13, 14]. The R package “EpiDISH” (method = “RPC”) was used to infer the fractions of a priori known cell subtypes present in blood specimens, using a whole blood reference of 333 tsDHS-DMCs (type specific DNAse hypersensitive site—differentially methylated CpGs) and seven blood cell subtypes (“centDHSbloodDMC.m”) [15, 16]. In addition, we used the LUMP algorithm (“rnb.execute.lump” function) within the R package “RnBeads” to estimate immune cell content to check for sample purity [17].

### Statistics

#### Pre- and post-chemotherapy cohort (paired samples)

Global differences in genomic DNAm levels, immune cell content, and cell type heterogeneity (CD19+ B cells, CD56+ natural killer cells, CD4+ T cells, CD8+ T cells, CD14+ monocytes, and granulocytes) were examined using paired samples Wilcoxon test. To identify differentially methylated CpG sites (probes) and promoter regions (promoters), linear regression models were fitted using the “limma*”* package in R and adjusted for monocytes in the paired sample analysis [18]. The outline of the design matrix is as follows: *model.matrix(*~ *0* + *time* + *serial* + *monocytes)*, where “time” refers to the specific time at which the sample was collected (i.e. pre- or post-chemotherapy). The term "serial" denotes the patient's unique study ID. To potentially increase statistical power and generate more interpretable sets of differentially methylated regions, we performed region-based analyses using definitions of promoters (1.5 kb upstream and 0.5 kb) downstream of the transcription start sites, Ensembl genes (v75) preloaded in “RnBeads”.

#### Gene set enrichment analysis

To identify key processes or gene sets that are differentially activated or suppressed between chemotherapy-treated and non-treated patients, we conducted gene set enrichment analysis using the R package “ClusterProfiler” (gseGO [ont = “BP”] and gseKEGG functions with parameters: exponent = 1, minGSSize = 10, maxGSSize = 500, eps = 0, pvalueCutoff = 1, pAdjustMethod = “none”, verbose = TRUE, use_internal_data = FALSE, seed = F, nPermSimple = 10,000, by = “fgsea”) [19]. Ranking metrics based on statistical significance (Z scores from promoters, i.e. systematic difference between treated and non-treated) from the linear regression models were used to rank genes [20]. Visualisation of the gene set enrichment analysis results in the form of dot plots was performed using the “enrichplot” package in R [19].

#### Validation of significant results in a single time point dataset (SGBCC)

We validated the probes and gene sets using the SGBCC dataset, following the same procedures as above. A deviation was in fitting the linear model using “limma”, as the SGBCC data is derived at a single time point. Here, we adjusted for batch, age at diagnosis, ethnicity, year of diagnosis, and immune cell content. Blood specimens were additionally adjusted for cell-type heterogeneity (CD19+ B cells, CD56+ natural killer cells, CD4+ T cells, CD8+ T cells, CD14+ monocytes, and granulocytes). Separate models were fitted for the dataset from blood specimens (*n* = 1808: 1,066 with chemotherapy and 742 without chemotherapy) and saliva specimens (*n* = 337: 207 with chemotherapy and 130 without chemotherapy).

In addition to the permutation of gene sets (nPermSimple = 10,000) within the gseGO and gseKEGG functions, we performed 500 permutations of the outcomes (chemotherapy yes and no) of the individuals within each dataset (blood and saliva) and repeated both the linear model fitting step and gene set enrichment analyses. P-value was calculated as the proportion of iterations that is more extreme than the actual enrichment score. Permutation of chemotherapy (yes/no) was done to test the null hypothesis that the set of genetic variants is not associated with chemotherapy.

#### Change in DNAm effect size over time from the start of chemotherapy

To study the potential recovery of DNAm effects (probes and promoters), we classified patients with chemotherapy based on the time between specimen collection and the start of chemotherapy (< 0.5 years, 0.5 to 2 years, > 2 years, unspecified). The outline of the design matrix is as follows: *model.matrix(*~ *0* + *chemotherapy* + *batch* + *age at diagnosis* + *ethnicity* + *immune cell content* + *cell type* [for blood specimens]*)*, where *chemotherapy* has five levels based on the time between specimen collection and the start of chemotherapy with the reference level as patients without chemotherapy. We repeated the gene set enrichment analysis focusing on gene sets that attained significance in the analysis of chemotherapy as a binary variable (yes vs no). The analysis was done for blood and saliva specimens separately.

## Results

### Characteristics of the 125 breast cancer patients with paired pre- and post-chemotherapy specimens

Table 1 shows a descriptive summary of the study population comprising subjects with paired samples. The median age at diagnosis of 125 breast cancer patients with DNAm information before and after chemotherapy was 56 years (interquartile range (IQR) 49–63). The majority were Chinese (*n* = 99 [79%]). The stage distribution at diagnosis was 16% (*n* = 20), 55% (*n* = 69), 28% (*n* = 35), and 1% (*n* = 1) for Stages I–IV, respectively. The majority of the patients completed anthracycline + taxane (*n* = 54, 43%), followed by taxane + carboplatin (*n* = 49, 39%) (Additional file 2: Table S3). We did not observe differences in global DNAm level (*p* = 0.902), and LUMP (*p* = 0.581) pre- and post-chemotherapy using the paired Wilcoxon test. Paired Wilcoxon tests on the proportion of cell types found monocytes (*p* = 0.001) and CD4+ T cells (*p* = 0.005) to be significantly changed after treatment (Additional file 2: Table S4). As the proportions of cell types sum to 1 for each individual and with our limited sample size, to avoid overfitting we adjusted only for monocytes (median increase in proportion after chemotherapy = 15%) in subsequent analyses.

**Table 1:** Characteristics of the 125 breast cancer patients with paired pre- and post-chemotherapy specimens

Variable	Statistics
Median age at diagnosis, years (IQR)	56 (49–63)
Ethnicity, *n* (%)	
Chinese	99 (79)
Non-chinese	26 (21)
Histology, *n* (%)	
Ductal	110 (88)
Lobular	8 (6)
Other	7 (6)
TNM stage, *n* (%)	
I	20 (16)
II	69 (55)
III	35 (28)
IV	1 (1)
ER status, *n* (%)	
Positive	83 (66)
Negative	42 (34)
PR status, *n* (%)	
Positive	74 (59)
Negative	51 (41)
HER2 status, *n* (%)	
Positive	55 (44)^a^
Negative	70 (56)
Genome-wide methylation, median (IQR)	0.655 (0.650–0.660)
Immune cell content (LUMP), median (IQR)	0.989 (0.988–0.990)
Cell type (proportion), median (IQR)	
CD19+ B-cell	0.004 (0.000–0.027)
Natural killer cell	0.012 (0.000–0.036)
CD4+ T cells	0.000 (0.000–0.035)
CD8+ T cells	0.090 (0.064–0.122)
Monocytes	0.061 (0.038–0.078)
Granulocytes	0.796 (0.690–0.860)

*IQR* interquartile range

^a^One patient refused targeted therapy

### Chemotherapy treatment was significantly associated with DNAm changes in 1568 probes and 164 promoters

**Fig. 1:**
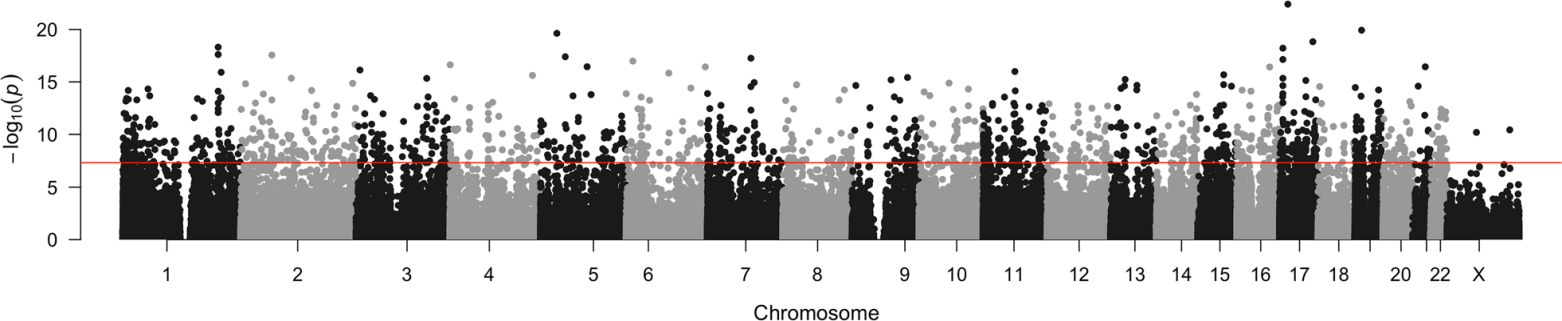
Manhattan plot of association results between chemotherapy and DNA methylation for 588,798 probes (125 breast cancer patients with pre- and post-chemotherapy paired samples), adjusted for the proportion of monocytes (*limma* design matrix ~ 0 + time + serial + monocytes). The term “time” refers to the specific time at which the sample was collected (i.e. pre- or post-chemotherapy). The term “serial” denotes the patient’s unique study ID. Using a Bonferroni threshold *p*-value < 8.49e-8, represented by a red line, a total of 1568 associations were found to be statistically significant (Additional file 3)

A total of 588,798 probes (chromosomes 1 through 22, and X) were retained after quality control. Adjusting for monocyte proportion, a total of 1,568 differentially methylated probes remained significantly associated with chemotherapy after adjustments and correction for multiple testing (*p* < 8.49e−8) (Additional file 3). Figure 1 shows the results of individual probes illustrated in a Manhattan plot. Regional analysis restricted to promoters showed 164 promoters to be significantly associated with chemotherapy (*p* < 1.21e−6) (Additional file 4).

### Gene set enrichment analysis identified suppressed biological processes and pathways related to sensory perception and smell

**Fig. 2:**
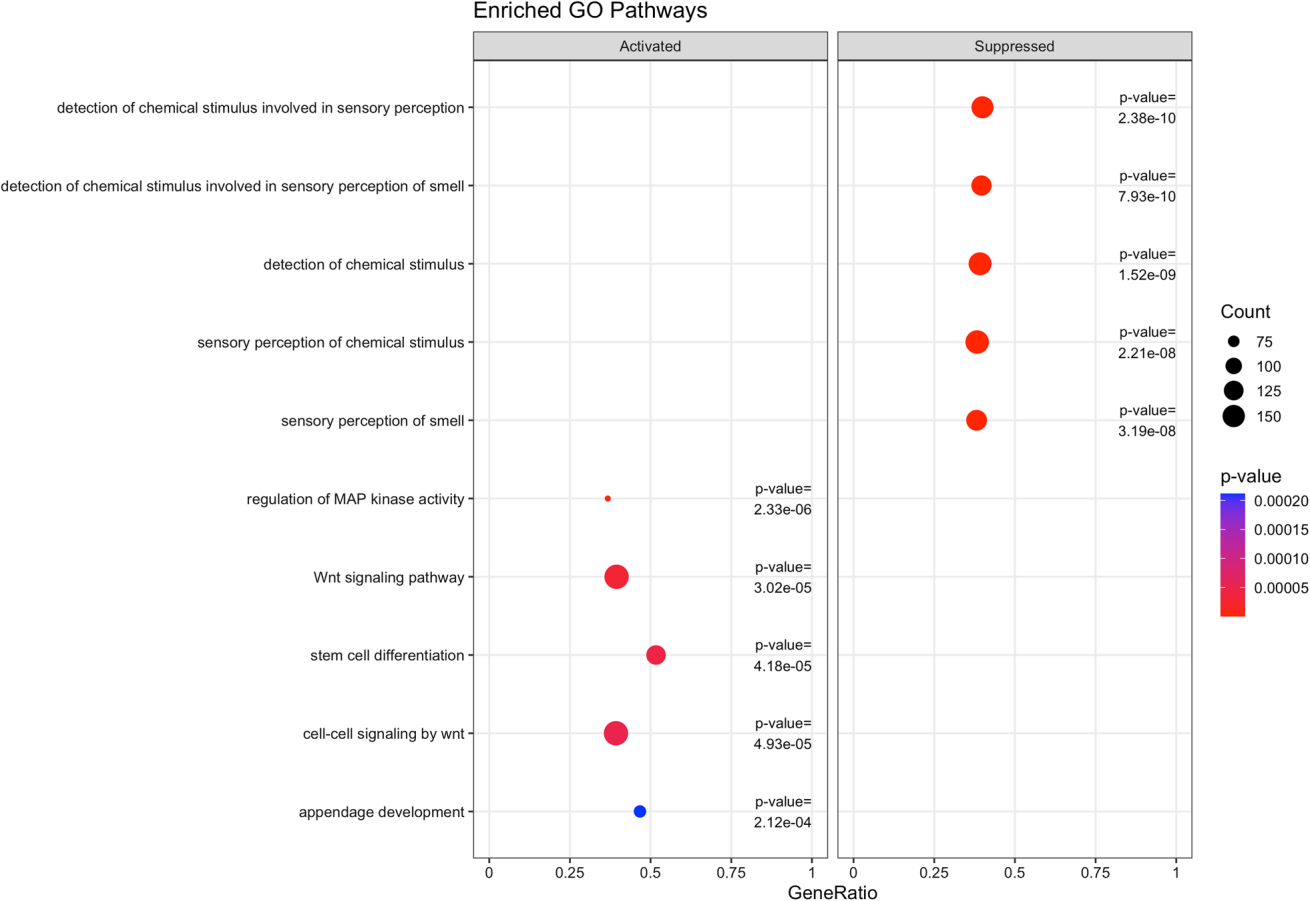
Top 5 activated and 5 suppressed enriched Gene Ontology (GO) biological processes using associations from adjusted models (*limma* design matrix ~ 0 + time + serial + monocytes), based on pre- and post-chemotherapy paired samples of 125 breast cancer patients. Count is the number of genes that belong to a given gene set

**Fig. 3:**
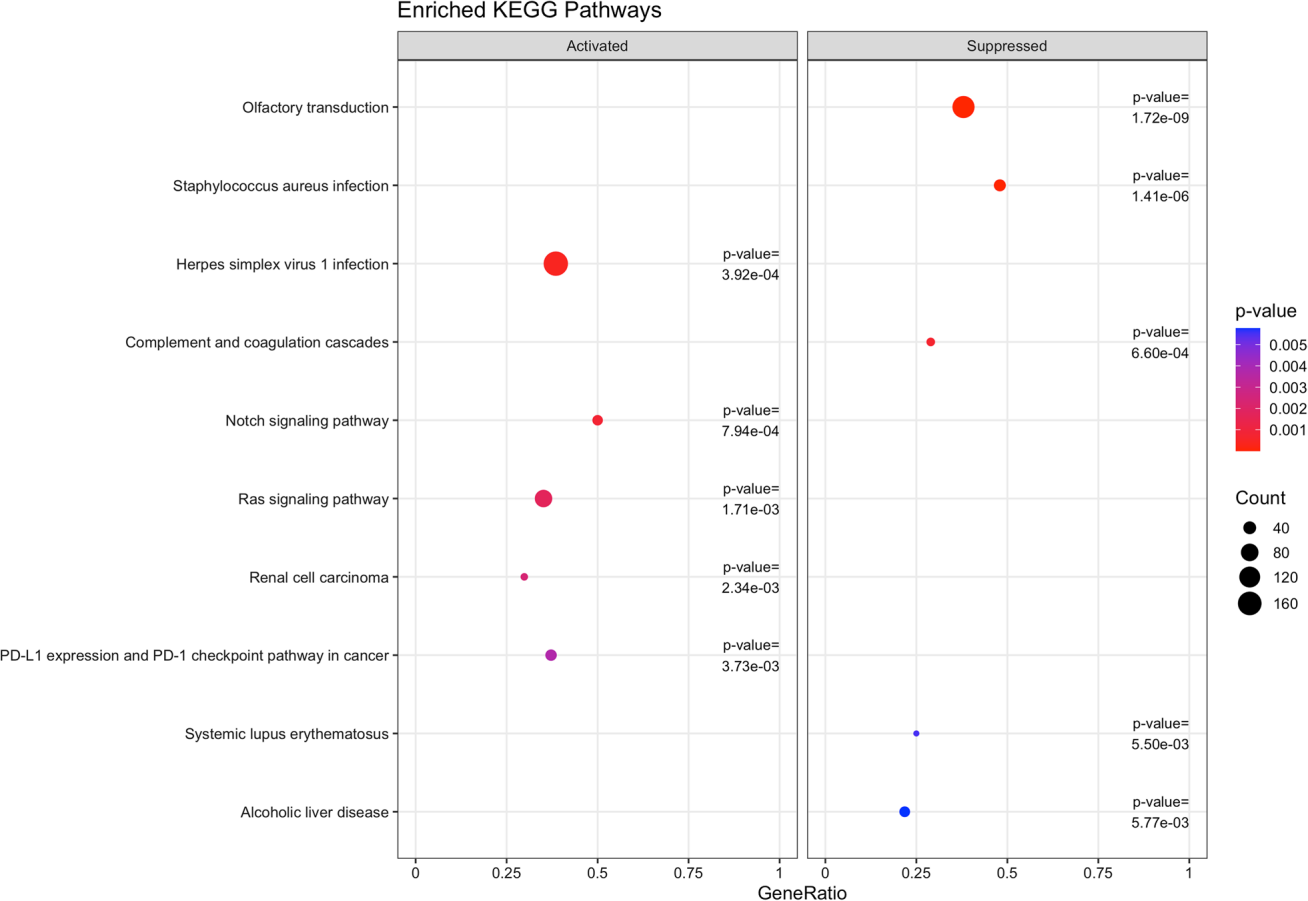
Top 5 activated and 5 suppressed enriched KEGG pathways using associations from adjusted models (*limma* design matrix ~ 0 + time + serial + monocytes), based on pre- and post-chemotherapy paired samples of 125 breast cancer patients. Count is the number of genes that belong to a given gene set

Gene set enrichment analysis on 41,207 promoters revealed six Gene Ontology (GO) terms associated with chemotherapy that survived Bonferroni correction (*p* < 1.21e−6) (GO:0007606, sensory perception of chemical stimulus; GO:0007608, sensory perception of smell; GO:0009593, detection of chemical stimulus; GO:0050906, detection of stimulus involved in sensory perception; GO:0050907, detection of chemical stimulus involved in sensory perception; and GO:0050911, detection of chemical stimulus involved in sensory perception of smell) (Fig. 2 and Additional file 5). One KEGG pathway (Olfactory transduction [hsa04740] *p* = 1.72e−9) was associated with chemotherapy (Fig. 3 and Additional file 6).

### Characteristics of the single time point validation dataset

The median age of the 2,145 breast cancer patients included in SGBCC was 49 years ([IQR] 44–57 years). Most were Chinese (*n* = 1808 [84%]), followed by 240 [11%] Malays, 87 [4%] Indians, and 10 [< 1%] of other or unknown ethnicity. No significant difference in global DNAm levels was observed between breast cancer patients treated and not-treated with chemotherapy (Kruskal Wallis test, *p* = 0.306) (Additional file 2: Table S5). DNA was extracted from blood specimens for 1808 patients (1066 treated with chemotherapy and 742 not-treated) and saliva specimens for 337 patients (207 treated with chemotherapy and 130 not treated). Characteristics of study participants are summarised in Additional file 2: Table S5 and Additional file 7.

### 141 CpG sites found to be significant in the paired sample analysis were also found to be associated with chemotherapy in the single time point dataset

A total of 525,100 probes (chromosomes 1 through 22, and X) were retained for analysis after quality control and filtering across specimen types (blood or saliva). Additional file 8: Figs. S1 and S2 show the results of individual probes illustrated in Manhattan plots by blood and saliva specimens, respectively (19,756 associations from blood and 389 associations from saliva specimens reached statistical significance [*p* < 9.52e−8]), adjusted for batch, immune cell content (LUMP), cell types (only for blood specimens), year of breast cancer diagnosis, and ethnicity (Additional files 9 and 10). We found 141 probes to be significantly associated (in the same direction) with chemotherapy in all three datasets (paired samples and single time point blood and saliva specimens) (Additional file 1: Fig. 2A and Additional file 2: Table S6).

### 11 promoters found to be significant in the paired sample analysis were also found to be associated with chemotherapy in the single time point dataset

Regional analysis restricted to promoters (*n* = 40,271, common across the five batches) showed 1485 genes from blood specimens and 50 from saliva specimens to be significantly associated with chemotherapy treatment (*p* < 1.24e−6), adjusted for batch, immune cell content (LUMP), cell types (only for blood specimens), year of breast cancer diagnosis, and ethnicity (Additional files 11 and 12). Eleven of these promoters (ENSG00000249526, ENSG00000262482, ENSG00000250616, ENSG00000207223, ENSG00000267610, ENSG00000260004, ENSG00000263847, and ENSG00000256083 mapped to RNA genes, ENSG00000213859 [*KCTD11*], and ENSG00000250984 [*COX6A1* pseudogene], ENSG00000106948 [*AKNA*]) were found to be significantly associated with chemotherapy in the paired samples dataset (Additional file 1: Fig. S2B and Additional file 2: Table S7).

### Gene set enrichment analysis in the single time point dataset validated suppressed biological processes and pathways related to sensory perception and smell

Gene set enrichment analysis on 40,271 promoters in the SGBCC dataset found the same six GO biological processes associated with chemotherapy treatment in both the blood and saliva datasets. (Additional file 8: Figs. S3 and S4, and Additional file 13). KEGG pathway “Olfactory transduction” (hsa04740) was also found to be significantly associated with chemotherapy treatment (*p*_blood_ = 2.03e−15 and *p*_saliva_ = 7.52e−56; adjusted for batch, LUMP, cell type, age at diagnosis, year of diagnosis, and ethnicity). The pathway remained significant when outcomes (chemotherapy yes/no) were permutated (*p*_blood_ = 0.028 and *p*_saliva_ = 0.007) (Additional file 8: Fig. S5 and S6, and Additional file 14).

**Fig. 4:**
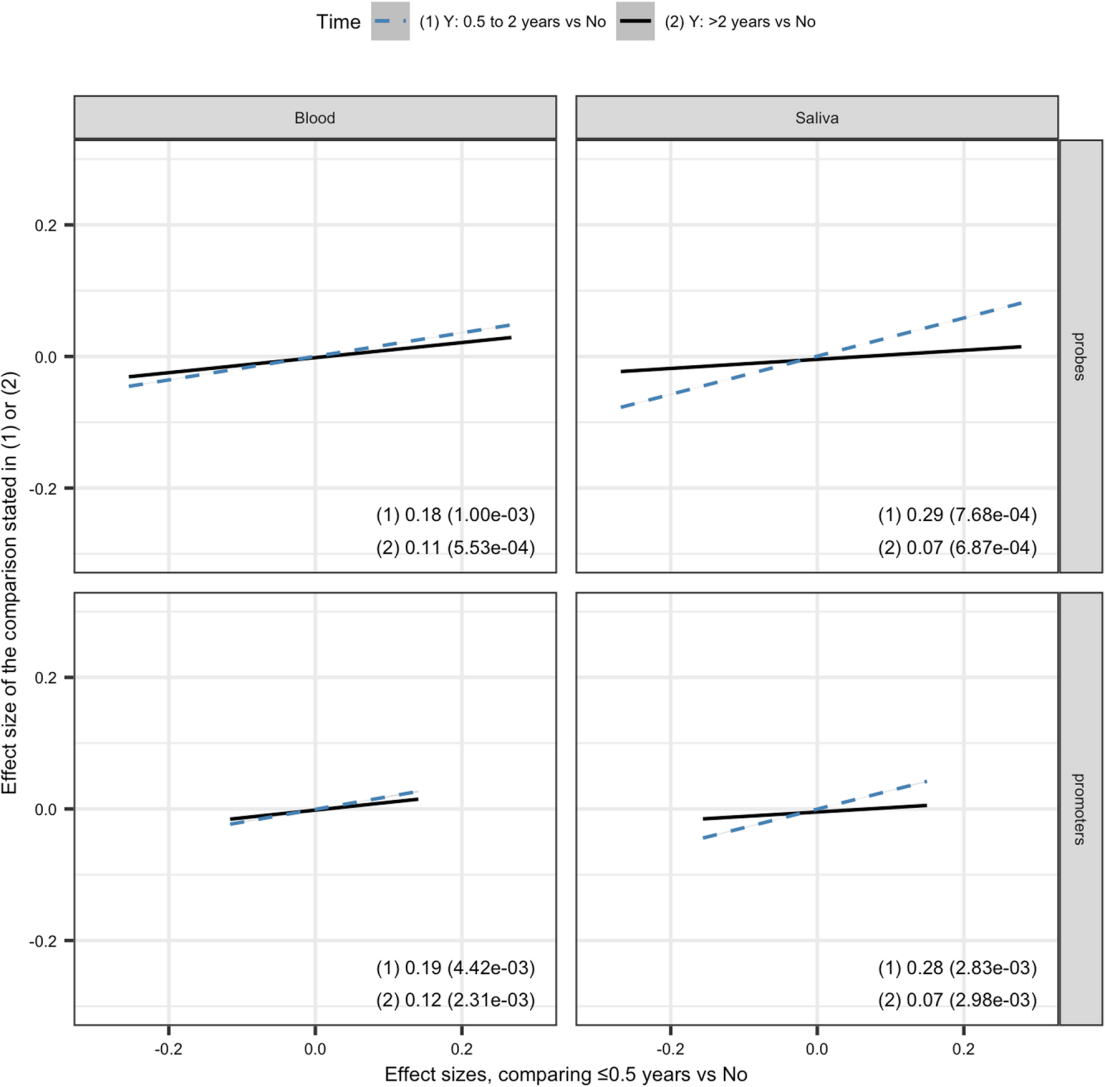
Comparing the strength of associations from the single time point analysis (effect size estimates from the linear model of the association between DNA methylation [525,100 probes and 40,271 promoters] and chemotherapy) by time since start of chemotherapy. The slopes and standard errors corresponding to each time comparison are shown in the bottom right of each panel

### The strength of associations decreased over time since the start of chemotherapy

Chemotherapy-treated patients with blood specimens (*n* = 1808) were subset into groups based on when the specimen was taken after chemotherapy: no chemotherapy (*n* = 742), within < 0.5 years (*n* = 203), 0.5–2 years (*n* = 113), and > 2 years (*n* = 338). The corresponding numbers of patients with saliva specimens are 130, 28, 32, and 76.

Comparing 203 patients with specimens taken < 0.5 years of chemotherapy to 742 non-chemotherapy treated patients with blood specimens [Comparison I], the number of significant differentially methylated probes was 27,083 (Additional file 8: Fig. S7, Additional file 15). The number of differentially methylated probes that remained significant (*p* < 9.52e−8) was reduced to 1,027 for blood specimens taken between 0.5 and 2 years [Comparison II], and further reduced to 221 for blood specimens taken > 2 years of chemotherapy [Comparison III] (Additional file 8: Figs. S8 and S9, Additional file 15). These 221 probes had associations in the same direction in all three comparisons, of which 101 (45%) probes’ effect size decreased across all three comparisons. We observed a decrease in effect size only in Comparison III (> 2 years vs no) from Comparison I (< 0.5 years vs no) in 95 (43%) probes.

Comparing 28 patients with specimens taken < 0.5 years of chemotherapy to 130 non-chemotherapy-treated patients with saliva specimens, the number of significant differentially methylated probes was 4555 (Additional file 8: Fig. S10, Additional file 15). The number of differentially methylated probes that remained significant (*p* < 9.52e−8) decreased to 51 for saliva specimens taken between 0.5 and 2 years and two for saliva specimens taken > 2 years of chemotherapy (Additional file 8: Figs. S11 and S12, Additional file 15). However, both probes were not significantly differentially methylated in blood specimens.

Figure 4 and Additional file 1: Fig. S3 show scatter plots of the effect size estimates obtained from comparisons of the three patient groups based on the time of specimen collection since chemotherapy against non-chemotherapy-treated patients. The effect size estimates for probes obtained from the 0.5–2 years after chemotherapy vs no chemotherapy comparison were generally smaller than that of the < 0.5 years after treatment vs no treatment comparison (slope = 0.18 for blood specimens, 0.29 for saliva specimens) (Additional file 15). The effect size estimates were more attenuated for the > 2 years after chemotherapy vs no chemotherapy comparison (slope = 0.11 for blood specimens, 0.07 for saliva specimens). Similar trends are observed for the promoter-level analyses (Additional file 1: Fig. S4 and Additional file 16).

Gene set enrichment analyses revealed the p-values associated with the KEGG pathway hsa04740 (Olfactory transduction) to be significant for the < 0.5 years after chemotherapy vs no chemotherapy comparison (*p* = 9.47e−20) for saliva specimens and the > 2 years after chemotherapy vs no chemotherapy comparison (*p* = 1.12e−15) (Additional file 1: Fig. S5). The pathway remained suppressed for all other comparison groups, with the exception of the > 2 years after chemotherapy vs no chemotherapy comparison for blood specimens.

## Discussion

Two datasets comprising a total of 2270 Asian breast cancer patients were used to study the effect of chemotherapy on the landscape of DNAm changes: (1) Patients with paired specimens collected pre- and post-chemotherapy, (2) single time point blood or saliva specimens from patients treated and not treated with chemotherapy. In addition, we found 141 differentially methylated CpGs and 11 promoters to be significantly associated with chemotherapy treatment in both the paired sample and single time point datasets after Bonferroni correction. Gene set enrichment analysis of promoters suggests an epigenetic basis by which chemotherapy treatment may affect the perception of smell. The effect size estimates obtained from the comparisons of treated vs non-treated samples generally decreased as the specimens were collected a longer time after the start of chemotherapy treatment, suggesting that chemotherapy-induced DNAm changes recover over time.

Results from our paired sample analysis validated Yao et al.’s work (93 paired pre- and post-treatment samples) which showed that the proportions of monocytes and CD4+ T cells estimated from DNAm data were significantly altered after treatment (3). However, no significant difference was found in the proportions of B cells in our study. In addition, we confirmed significant chemotherapy-associated DNAm changes for cg16936953 in *TMEM49*/*VMP1/MIR21* (*p* = 6.08e−10), cg01252023 in *CORO1B* (*p* = 1.02e−16), and cg19956914 in *SUMF2* (*p* = 3.30e−11). cg11859398 in *SDK1* did not survive Bonferroni correction in our study.

Apart from CpG probes, we examined promoter regions as these may have distinct functional and regulatory roles. Eight of the eleven differentially methylated promoters that were significant across both datasets mapped to RNA genes. Although such genes have been suggested to play a role in the regulation of the olfactory system, their functions are unclear [21]. *KCTD11* plays a role as a marker and a regulator of neuronal differentiation. *AKNA* acts as a transcription factor that specifically activates the expression of the *CD40* receptor and its ligand *CD40L/CD154*, two cell surface molecules on lymphocytes that are critical for antigen-dependent B-cell development [22].

Our promoter-level pathway analysis revealed that biological processes related to sensory perception and the olfactory transduction pathway are significantly altered by chemotherapy. The single time point dataset additionally revealed the KEGG pathway “Taste transduction” (hsa04742) to be suppressed in chemotherapy-treated patients. Although the taste-related pathway did not survive stringent Bonferroni correction, the *p*-values in the various models tested across blood and saliva specimens ranged from 0.0005 to 0.006, and the corresponding less-stringent false-discovery rate Q values from 0.002 to 0.133.

Common side effects of chemotherapy include unwanted changes in taste and smell, which have repercussions on nutritional status, dietary intake, appetite, body mass index, and quality of life [23, 24]. It has been reported that chemosensory alterations occur in as many as 86% of chemotherapy-treated patients [25, 26]. The biological mechanisms driving such taste and smell disturbances are unclear [27].

The prevailing explanations include cytotoxic damage to proliferative olfactory and gustatory receptor cells, changes to oral microbiota, mucositis, nutritional imbalance, and alterations of salivary quantity and composition [27, 28]. However, previous studies have shown that sensory variability in taste and smell may be driven by epigenetic markers in non-cancer cohorts [29, 30]. As sensory changes are typically transient and mostly recover to baseline levels after cessation of treatment, a modifiable epigenetic mechanism such as DNAm is highly possible [31, 32]. In agreement, our results support the hypothesis that DNAm influences sensory factors to cause dysfunctions in olfactory measures, in chemotherapy-treated breast cancer patients. The attenuation of effect sizes of differentially methylated probes in our results supports clinical observations of recovery of the phenotype over time.

One notable caveat of the striking pathway analysis results is that the olfactory gene family is large, and genes related to olfactory receptors tend to be clustered in the genome, which can lead to spurious enrichment in gene ontology tests [33, 34]. However, Lerm et al. revealed that long-lasting alterations to DNAm patterns induced by SARS-CoV-2 infection are associated with negative impacts on odor perception, corroborating our finding that smell may be epigenetically rewired [35].

This is the first epigenome-wide study describing associations between chemotherapy treatment and genomic DNAm in a sizable cohort of Asian breast cancer patients. The convergence of findings using two separate study designs (paired sample pre- and post-chemotherapy and data obtained from a large cohort of chemotherapy-treated and non-treated patients) increases the rigor and validity of the study. The genome-wide differential methylation screening approach allows an unbiased selection of DNAm markers, which constitutes the main strength of our study.

It is important to acknowledge that the process of prioritizing samples for inclusion in DNAm experiments based on the completeness of clinical and follow-up data could potentially introduce a selection bias. In particular, included patients were more recently diagnosis, of younger age at diagnosis, and had a shorter time between sample collection and the start of chemotherapy (Additional file 17). Efforts should be made to minimize missing data and ensure comprehensive data collection to reduce the potential for selection bias in future studies. In addition, surgery is a major stress event for patients and has been shown to cause changes in DNAm [36]. However, the number of neoadjuvant breast cancer patients who received chemotherapy before surgery comprises only a quarter of our patient population. The low sample size limits our ability to examine surgery as a potential confounder. Further studies with a larger sample size will be required to study the effects by specific chemotherapy regimens. Due to the retrospective nature of the study, DNA samples were stored for different periods before processing for the DNAm experiments. Variation in DNAm stability over time in different genomic loci is likely [37]. DNAm is also tissue- and cell-specific, limiting the generalizability of the results to the sample types studied [38, 39]. The time between blood draw and the start of chemotherapy was thus taken into account in the analyses. The underlying distributions of DNAm data do not always satisfy the assumptions of linear regression models used for the analyses. However, this statistical method is valid for exploratory studies [40]. As data on body composition and behavioral risk factors such as dietary intake, physical activity, smoking, and drinking after breast cancer diagnosis were not collected, mediation analysis was not conducted.

## Conclusions

In conclusion, we found no significant difference in global genomic DNAm associated with chemotherapy in Asian breast cancer patients. However, individual CpG sites and promoter regions were observed to be differentially methylated. The enrichment of imprinted genes within biological processes and pathways suggests an epigenetic change by which chemotherapy could affect the perceptions of smell.

### Supplementary Information

**Additional file 1**. Supplementary Figures 1 to 5.

**Additional file 2**. Supplementary Tables 1 to 7.

**Additional file 3**. Association between chemotherapy and DNA methylation in CpG probes (pre- and post-chemotherapy paired samples in 125 breast cancer patients). Presented here are associations that reached statistical significance at a Bonferroni threshold p-value of <8.49e-8. Standard error = stdev.unscaled * s2.post.

**Additional file 4**. Association between chemotherapy and DNA methylation in promoter regions (pre- and post-chemotherapy paired samples in 125 breast cancer patients). Presented here are associations that reached statistical significance at a Bonferroni threshold p-value of <1.21e-6. Standard error = stdev.unscaled * s2.post.

**Additional file 5**. Gene set enrichment analysis of Gene Ontology (GO) biological processes from results of unadjusted and adjusted models in paired pre- and post-chemotherapy samples of 125 breast cancer patients who received chemotherapy.

**Additional file 6**. Gene set enrichment analysis of KEGG pathways from results of unadjusted and adjusted models in paired pre- and post-chemotherapy samples of 125 breast cancer patients who received chemotherapy.

**Additional file 7**. Characteristics of 2,145 breast cancer patients in the Singapore Breast Cancer Cohort (single time point analysis). P-values are from the Chi-square test and Wilcoxon test for categorical and continuous variables, respectively. We excluded unknown values when performing the tests.

**Additional file 8**. Additional file - Figures 1 to 12.

**Additional file 9.** Association between chemotherapy and DNA methylation in 525,100 CpG probes in 1,808 breast cancer patients with blood specimens (1,066 with chemotherapy and 742 without chemotherapy). Presented here are associations that reached statistical significance at a Bonferroni threshold p-value (p<9.52e-8) in either unadjusted analysis or after adjustments for batch, immune cell content (LUMP), cell types, year of breast cancer diagnosis, and ethnicity. Standard error = stdev.unscaled * s2.post.

**Additional file 10**. Association between chemotherapy and DNA methylation in 525,100 CpG probes in 337 breast cancer patients with saliva specimens (207 with chemotherapy and 130 without chemotherapy). Presented here are associations that reached statistical significance at a Bonferroni threshold p-value (p<9.52e-8) in either unadjusted analysis or after adjustments for batch, immune cell content (LUMP), year of breast cancer diagnosis, and ethnicity. Standard error = stdev.unscaled * s2.post.

**Additional file 11**. Association between chemotherapy and DNA methylation in 40,271 promoter regions in 1,808 breast cancer patients with blood specimens (1,066 treated and 742 not treated with chemotherapy). Presented here are associations that reached statistical significance at a Bonferroni threshold p-value (p<1.24e-6) in either unadjusted analysis or after adjustments for batch, immune cell content (LUMP), cell types, year of breast cancer diagnosis, and ethnicity. Standard error = stdev.unscaled * s2.post.

**Additional file 12**. Association between chemotherapy and DNA methylation in 40,271 promoter regions in 337 breast cancer patients with saliva specimens (207 treated and 130 not treated with chemotherapy). Presented here are associations that reached statistical significance at a Bonferroni threshold p-value (p<1.24e-6) in either unadjusted analysis or after adjustments for batch, immune cell content (LUMP), year of breast cancer diagnosis, and ethnicity. Standard error = stdev.unscaled * s2.post.

**Additional file 13**. Gene set enrichment analysis of Gene Ontology (GO) biological processes from results of unadjusted and adjusted models, by specimen type (blood or saliva).

**Additional file 14**. Gene set enrichment analysis of KEGG pathways using results from unadjusted and adjusted models, by specimen type (blood or saliva).

**Additional file 15**. Subset analysis, by time between chemotherapy start date and sample taken, of the association between chemotherapy and DNA methylation in 525,100 CpG probes in 1,808 breast cancer patients with blood specimens (1,066 treated and 742 not treated with chemotherapy) and 337 breast cancer patients with saliva specimens (207 treated and 130 not treated with chemotherapy). Presented here are associations that reached statistical significance at a Bonferroni threshold p-value (p<9.52e-8) with adjustments for batch, immune cell content (LUMP), cell types (only for blood), year of breast cancer diagnosis, and ethnicity. Standard error = stdev.unscaled * s2.post.

**Additional file 16**. Subset analysis, by time between chemotherapy start date and sample taken, of the association between chemotherapy and DNA methylation in 40,271 promoter regions in 1,808 breast cancer patients with blood specimens (1,066 treated and 742 not treated with chemotherapy) and 337 breast cancer patients with saliva specimens (207 treated and 130 not treated with chemotherapy). Presented here are associations that reached statistical significance at a Bonferroni threshold p-value (p<9.52e-8) within adjustments for batch, immune cell content (LUMP), cell types (only for blood), year of breast cancer diagnosis, and ethnicity. Standard error = stdev.unscaled * s2.post.

**Additional file 17**. Characteristics of the 7,177 source cohort's (the Singapore Breast Cancer Cohort) patients with blood or saliva samples and clinical data. Comparison to the 2,145 selected patients in this study was done using the Chi-square test for categorical variables and the Kruskal Wallis test for the continuous variables, unknown values were excluded from the test.

## Data Availability

The datasets used and/or analysed during the current study are available from the corresponding author upon reasonable request.

## Abbreviations

5mC: 5-Methylcytosines
BMIQ: Beta-mixture quantile
CpG: Cytosines that precede a guanine nucleotide
DNAm: DNA methylation
GO: Gene ontology
IQR: Interquartile range
KEGG: Kyoto encyclopedia of genes and genomes
LUMP: Leukocytes unmethylation for purity
SGBCC: Singapore Breast Cancer Cohort
SNP: Single nucleotide polymorphisms
tsDHS-DMCs: Type-specific DNAse hypersensitive site-differentially methylated CpGs
