# Supplementary material for: Alterations to DNA methylation patterns induced by chemotherapy treatment are associated with negative impacts on the olfactory pathway
Source: Breast Cancer Res. 2023 Nov 6;25:136. doi: 10.1186/s13058-023-01730-4 (PMC10626732; doi:10.1186/s13058-023-01730-4)

**Supplementary Figure 1.** Flowchart of how the analytical cohort was derived in the single time point analysis.

|  |  |  |  |  |  |
| --- | --- | --- | --- | --- | --- |
|  | **Number of patients with blood or saliva specimens** | |  |  |  |
|  | n=7,305 | |  |  |  |
|  |  |  |  | **Number of unselected specimens** |  |
|  |  |  |  | n=4,941 |  |
|  | **Number of speciments used in methylation experiment** | |  |  |  |
|  | n=2,364 | |  |  |  |
|  |  |  |  | **Exclusion (total n=118)**  Duplicated specimens (n=9)  Fail quality control |  |
|  |  |  |  | - Negative (n=50) |  |
|  |  |  |  | - Specificity I (n=13) |  |
|  |  |  |  | - Staining (n=6) |  |
|  |  |  |  | - Greedycut (n=5) |  |
|  |  |  |  | - Bisulfite conversion I (n=3) |  |
|  |  |  |  | - Sample type unknown (n=32) |  |
|  | **Number of unique patients that passed quality check** | |  |  |  |
|  | n=2,246 | |  |  |  |
|  |  |  |  | **Exclusion (total n=101)** |  |
|  |  |  |  | LUMP<0.9 in blood specimens (n=3) |  |
|  |  |  |  | Unknown chemotherapy status (n=98) |  |
|  | **Number of patients in analytical data** | |  |  |  |
|  | n=2,145 | |  |  |  |
|  |  |  |  |  |  |

**Supplementary Figure 2**. Probes and promoters significant after Bonferroni correction across the three datasets (paired samples, SGBCC blood specimens, and SGBCC saliva specimens).

**
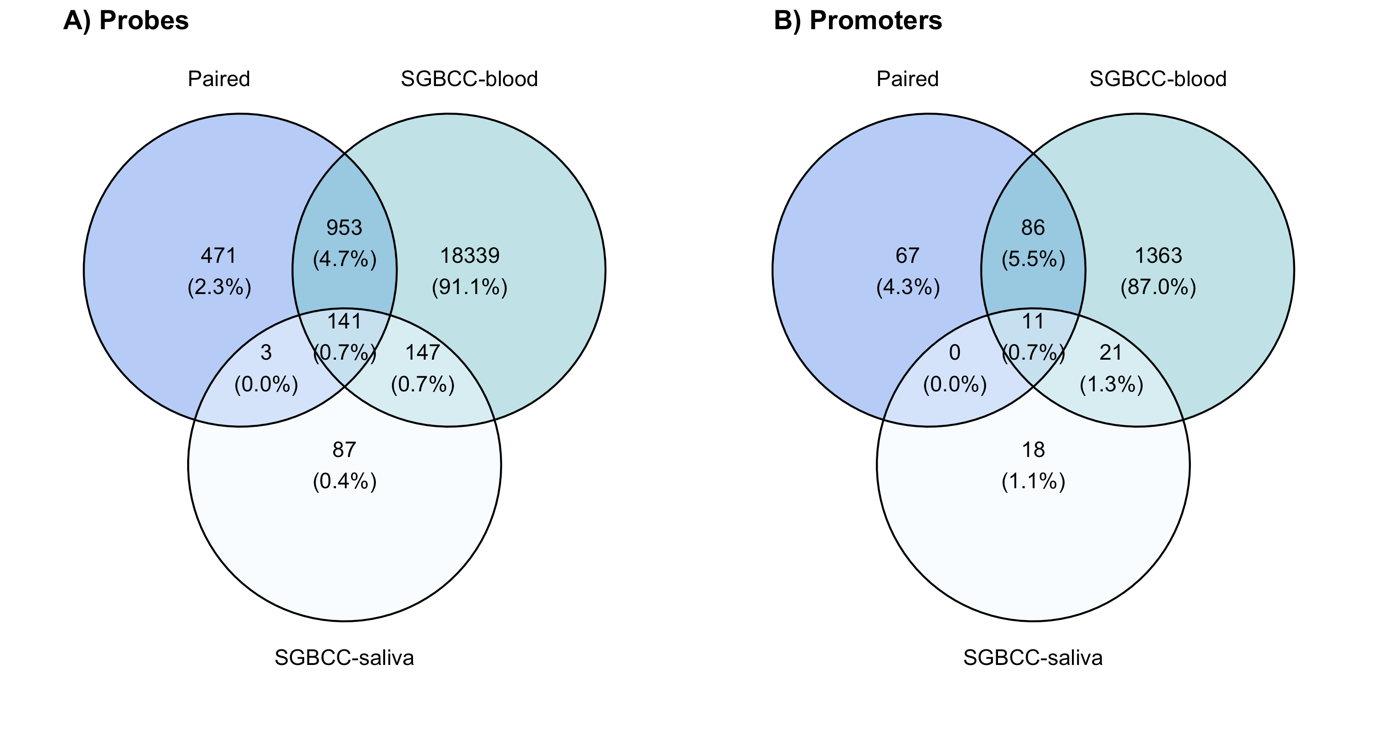
**

**Supplementary Figure 3**. Scatterplots comparing strength of associations from the single time point analysis (effect sizes from the linear model of the association between DNA methylation [525,100 probes] and chemotherapy) by time since start of chemotherapy. The slopes and standard errors [SE] corresponding to each time comparison are shown in the bottom right of each panel.


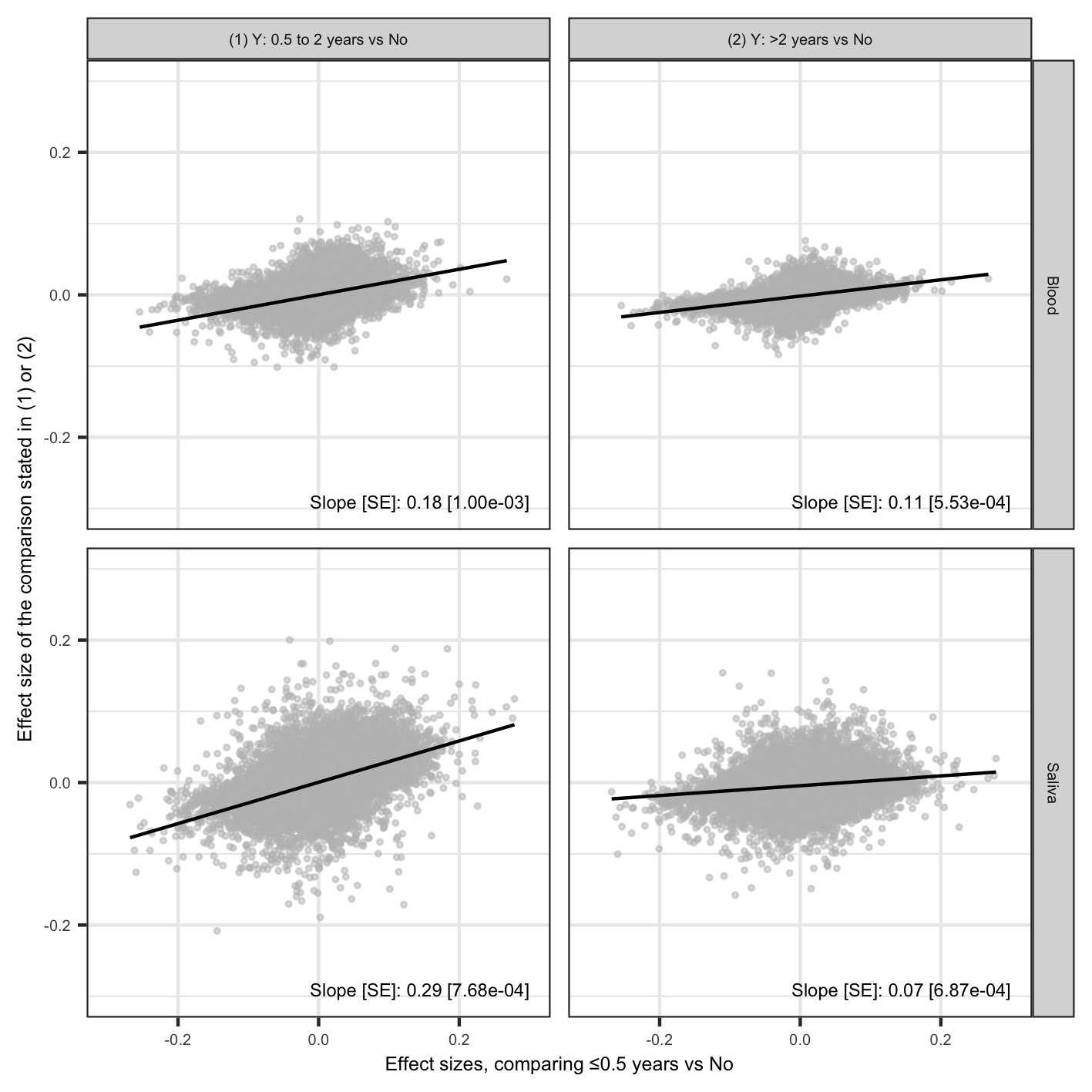


**Supplementary Figure 4**. Scatterplots comparing strength of associations from the single time point analysis (effect sizes from the linear model of the association between DNA methylation [40,271 promoters] and chemotherapy) by time since start of chemotherapy. The slopes and standard errors [SE] corresponding to each time comparison are shown in the bottom right of each panel.


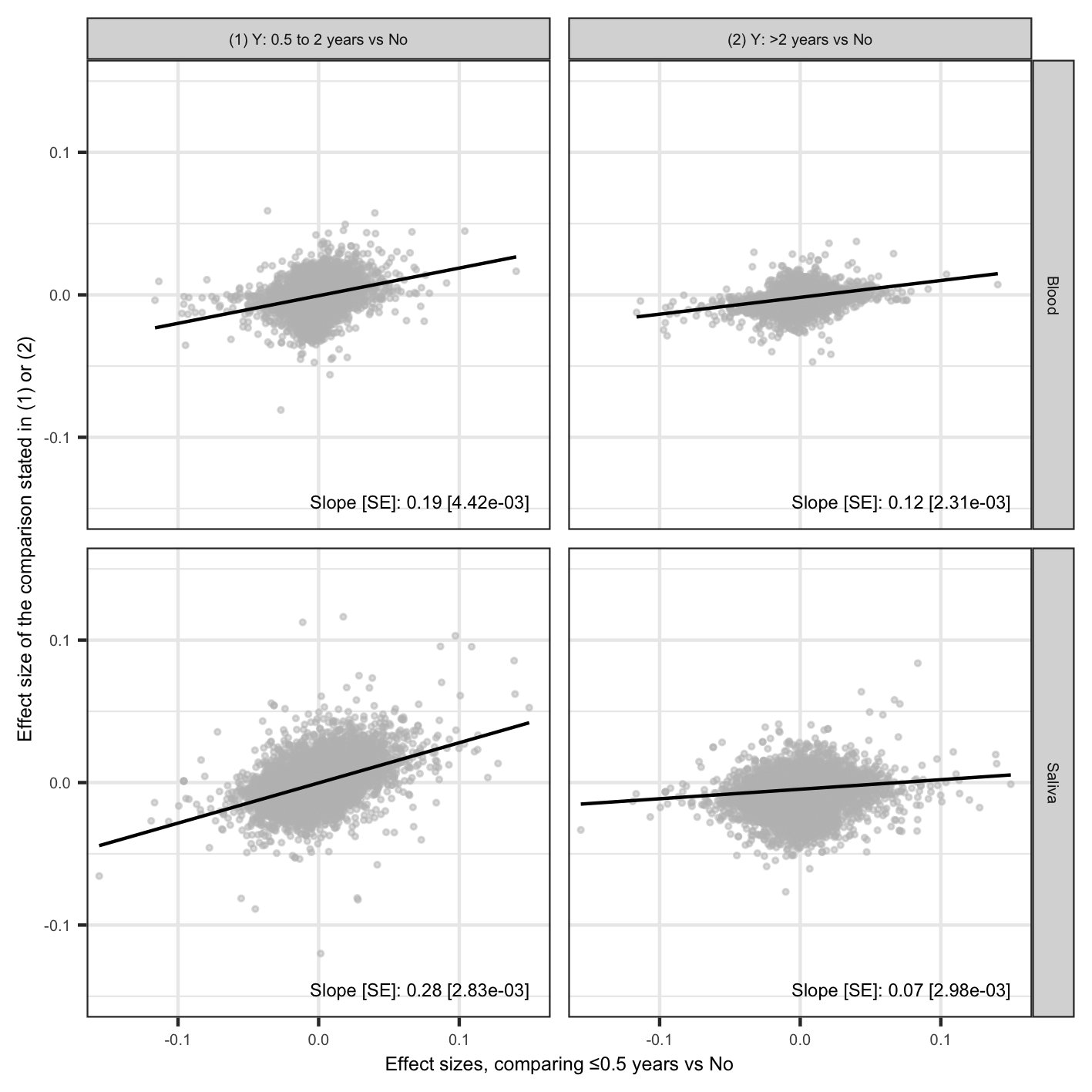


**Supplementary Figure 5**. Olfactory transduction pathway (enriched KEGG pathways) in SGBCC dataset, by sample type (blood or saliva). Adjustments: (1) blood – batch, age at diagnosis, cell types, LUMP, ethnicity, and year of diagnosis; (2) saliva – batch, age at diagnosis, LUMP, ethnicity, and year of diagnosis; (3) blood – batch, age at diagnosis, cell types, LUMP, and ethnicity; (4) saliva – batch, age at diagnosis, LUMP, and ethnicity.


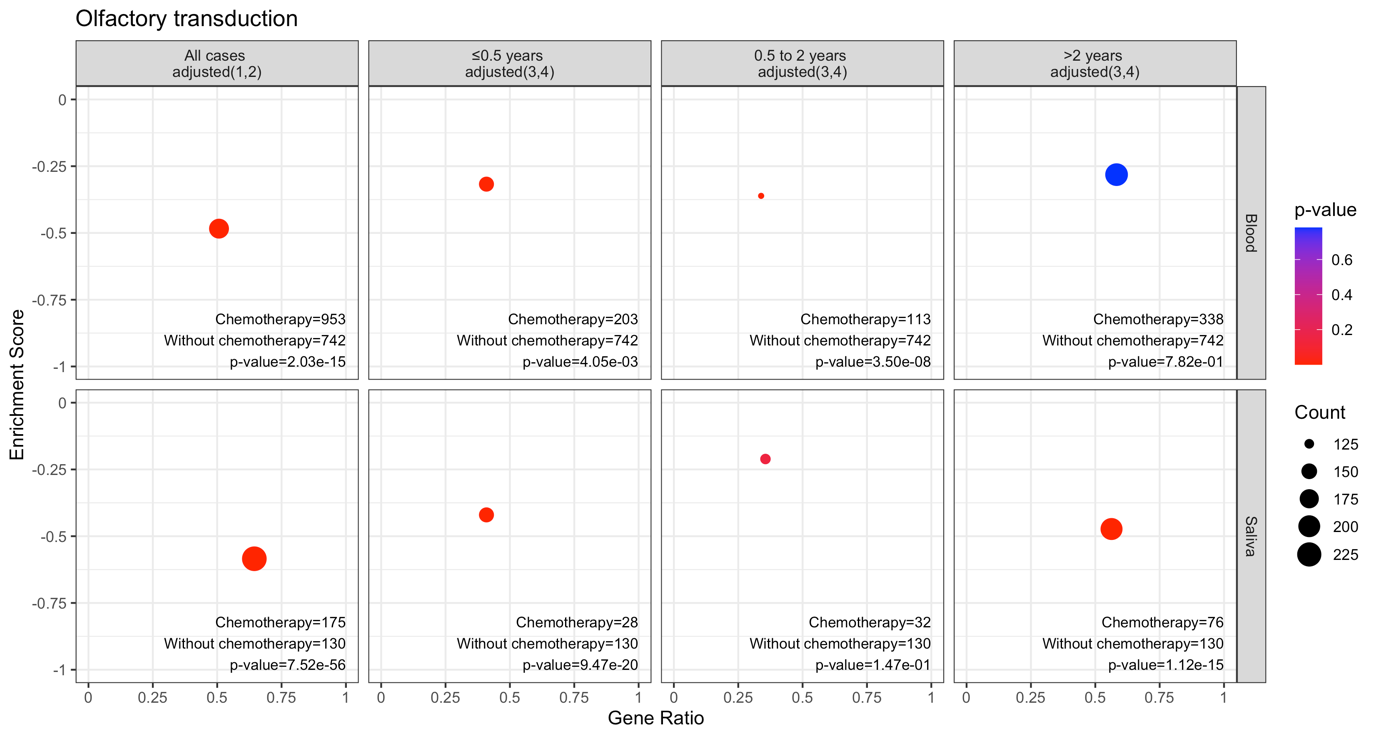

Supplement: Supplementary file 1 — Additional file 1. Supplementary Figures 1 to 5. [file 13058_2023_1730_MOESM1_ESM.docx]
