# Supplementary material for: Alterations to DNA methylation patterns induced by chemotherapy treatment are associated with negative impacts on the olfactory pathway
Source: Breast Cancer Res. 2023 Nov 6;25:136. doi: 10.1186/s13058-023-01730-4 (PMC10626732; doi:10.1186/s13058-023-01730-4)
Supplement: Supplementary file 2 — Additional file 2. Supplementary Tables 1 to 7. [file 13058_2023_1730_MOESM2_ESM.docx]

**Supplementary Table 1**. Options of the executed pre-processing module in RnBeads.

| **Option** | **Value** |
| --- | --- |
| filtering.whitelist |  |
| filtering.blacklist |  |
| filtering.snp | any |
| filtering.cross.reactive | yes |
| filtering.greedycut | yes |
| filtering.greedycut.pvalue.threshold | 0.01 |
| filtering.greedycut.rc.ties | row |
| imputation.method | knn |
| normalization.method | bmiq |
| normalization.background.method | enmix.oob |
| normalization.plot.shifts | yes |
| filtering.context.removal | CC, CAG, CAH, CTG, CTH, Other |
| filtering.missing.value.quantile | 0.9 |
| filtering.sex.chromosomes.removal | no |
| filtering.deviation.threshold | 0.005 |
| distribution.subsample | 1000000 |

**Supplementary Table 2.** Description of probes across six datasets which were processed and normalized independently. 525,100 probes and 40,271 promoters were common across the five batches from the Singapore Breast Cancer Cohort.

|  |  | **Singapore Breast Cancer Cohort** | | | | |
| --- | --- | --- | --- | --- | --- | --- |
|  | **Paired samples** | **Blood 1** | **Blood 2** | **Blood 3** | **Saliva 1** | **Saliva 2** |
| SNP-enriched probes removed | 139,721 | 139,721 | 139,721 | 139,721 | 139,721 | 139,721 |
| Cross-reactive probes removed | 34,264 | 34,264 | 34,264 | 34,264 | 34,264 | 34,264 |
| Probes removed by Greedycut | 10,410 | 12,314 | 20,145 | 12,516 | 11,988 | 8,932 |
| Context-specific probes removed | 1,166 | 1,142 | 1,067 | 1,131 | 1,125 | 1,174 |
| Consistent probes removed (standard deviation lower than 0.005) | 92,514 | 108,923 | 98,247 | 101,366 | 91,807 | 92,962 |
| Y chromosome probes removed | 22 | 28 | 24 | 20 | 29 | 26 |
| Total probes retained | 588,798 | 573,423 | 570,507 | 577,877 | 587,961 | 589,816 |
| Total promoters retained | 41,207 | 40,882 | 40,903 | 41,154 | 41,211 | 41,335 |

**Supplementary Table 3**. Completed chemotherapy regimens in paired pre- and post-chemotherapy samples of 125 breast cancer patients who received chemotherapy.

| **Anthracycline** | **Dose dense anthracycline** | **Taxane** | **Carboplatin** | **n (%)** |
| --- | --- | --- | --- | --- |
| Y | N | N | N | 3 (2) |
| Y | Y | N | N | 2 (2) |
| N | N | Y | N | 10 (8) |
| Y | N | Y | N | 54 (43) |
| Y | Y | Y | N | 5 (4) |
| N | N | Y | Y | 49 (39) |
| Y | Y | Y | Y | 2 (2) |

**Supplementary Table 4.** Median (interquartile range) cell type proportions pre- and post-chemotherapy and median difference in cell type proportions after chemotherapy in 125 breast cancer patients. Proportions sum to 1 for each individual. Wilcoxon paired test was used to test for change in cell type proportion. IQR: interquartile range

| **Cell type** | **Pre- (IQR)** | **Post- (IQR)** | **Difference (IQR)** | **P-value** |
| --- | --- | --- | --- | --- |
| CD19+ B-cell | 0.004  (0.000 to 0.027) | 0.000  (0.000 to 0.028) | 0.000 (-0.009 to 0.009) | 0.870 |
| Natural killer cell | 0.012  (0.000 to 0.036) | 0.013  (0.000 to 0.046) | 0.000 (-0.015 to 0.026) | 0.378 |
| CD4+ T cells | 0.000 (0.000 to 0.035) | 0.000  (0.000 to 0.022) | 0.000 (-0.018 to 0.000) | 0.005 |
| CD8+ T cells | 0.090  (0.064 to 0.122) | 0.100  (0.066 to 0.136) | 0.008 (-0.026 to 0.038) | 0.117 |
| Monocytes | 0.061  (0.038 to 0.078) | 0.068  (0.055 to 0.090) | 0.008 (-0.014 to 0.036) | 0.001 |
| Granulocytes | 0.796  (0.690 to 0.860) | 0.768  (0.712 to 0.831) | -0.017 (-0.095 to 0.058) | 0.194 |

**Supplementary Table 5.** Description of 2,145 breast cancer patients in the Singapore Breast Cancer Cohort (single time point analysis) by chemotherapy status at time of specimen collection. IQR: interquartile range.

|  | **Chemotherapy before specimen collection** | |  |
| --- | --- | --- | --- |
|  | **No** | **Yes** | **p** |
|  | **n=872** | **n=1273** |  |
| **Median self-reported age at diagnosis, years (IQR)** | 51.0 (46.0 to 61.2) | 47.0 (43.0 to 54.0) | 7.61E-26 |
| **Ethnicity (%)** |  |  |  |
| Chinese | 745 (85) | 1063 (84) | 5.22E-01 |
| Malay | 92 (11) | 148 (12) |  |
| Indian | 32 (4) | 55 (4) |  |
| Other/ unknown | 3 (0) | 7 (1) |  |
| **Sample type** |  |  |  |
| Blood | 742 (85) | 1066 (84) | 4.32e-01 |
| Saliva | 130 (15) | 207 (16) |  |
| **Median time between sample collection and diagnosis, years (IQR)** | 0.2 (0.1 to 1.0) | 2.0 (0.4 to 6.5) | 1.20E-80 |
| *Unknown, n* | *1* | *5* |  |
| **Median time between sample collection and start of chemotherapy, years (IQR)** | -0.1 (-0.2 to 0.0) | 2.3 (0.4 to 6.0) | 2.69E-127 |
| *No chemotherapy/ undetermined dates/, n* | *618* | *483* |  |
| **Median genome-wide methylation (IQR)** | 0.654 (0.646 to 0.660) | 0.654 (0.647 to 0.660) | 3.06E-01 |
| **Median immune cell content, LUMP (IQR)** | 0.984 (0.981 to 0.986) | 0.984 (0.980 to 0.986) | 8.95E-02 |
| **Stage (%)** |  |  |  |
| 0 | 54 (6) | 3 (0) | 5.03E-61 |
| I | 363 (42) | 223 (18) |  |
| II | 194 (22) | 531 (42) |  |
| III | 66 (8) | 247 (19) |  |
| IV | 52 (6) | 68 (5) |  |
| Unknown | 143 (16) | 201 (16) |  |
| **Nodal status (%)** |  |  |  |
| Positive | 175 (20) | 504 (40) | 1.04E-30 |
| Negative | 628 (72) | 540 (43) |  |
| Unknown | 69 (8) | 221 (17) |  |
| **Metastatic status (%)** |  |  |  |
| No | 719 (82) | 1146 (90) | 3.45E-01 |
| Yes | 52 (6) | 68 (5) |  |
| Unknown | 101 (12) | 59 (5) |  |
| **Grade (%)** |  |  |  |
| Well-differentiated | 195 (22) | 101 (8) | 5.16E-26 |
| Moderate-differentiated | 338 (39) | 468 (37) |  |
| Poorly-differentiated | 277 (32) | 620 (49) |  |
| Unknown | 62 (7) | 86 (7) |  |
| **ER-status (%)** |  |  |  |
| Positive | 674 (77) | 853 (67) | 2.37E-09 |
| Negative | 162 (19) | 388 (30) |  |
| Unknown | 36 (4) | 32 (2) |  |
| **PR-status (%)** |  |  |  |
| Positive | 627 (72) | 795 (62) | 4.55E-08 |
| Negative | 205 (24) | 443 (35) |  |
| Unknown | 40 (5) | 35 (3) |  |
| **HER2-status (%)** |  |  |  |
| Positive | 177 (20) | 348 (27) | 6.76E-04 |
| Negative | 551 (63) | 749 (59) |  |
| Unknown | 144 (17) | 176 (14) |  |
| **Surgery (%)** |  |  |  |
| No | 38 (4) | 25 (2) | 2.60E-03 |
| Yes | 812 (93) | 1191 (94) |  |
| Unknown | 22 (3) | 57 (4) |  |
| **Radiotherapy (%)** |  |  |  |
| No | 429 (49) | 359 (28) | 1.40E-24 |
| Yes | 380 (44) | 832 (65) |  |
| Unknown | 63 (7) | 82 (6) |  |
| **Endocrine therapy (%)** |  |  |  |
| No | 283 (32) | 312 (25) | 9.00E-06 |
| Yes | 579 (66) | 921 (72) |  |
| Unknown | 10 (1) | 40 (3) |  |
| **Cell type proportion (n=1,817 blood specimens), median (IQR)** |  |  |  |
| CD19+ B-cell | 0.019 (0.000 to 0.040) | 0.021 (0.000 to 0.053) | 2.22E-01 |
| Natural killer cell | 0.020 (0.000 to 0.053) | 0.021 (0.000 to 0.058) | 2.84E-01 |
| CD4+ T cells | 0.001 (0.000 to 0.034) | 0.000 (0.000 to 0.023) | 1.23E-04 |
| CD8+ T cells | 0.103 (0.073 to 0.137) | 0.110 (0.074 to 0.146) | 4.05E-02 |
| Monocytes | 0.078 (0.063 to 0.096) | 0.087 (0.070 to 0.112) | 7.27E-12 |
| Granulocytes | 0.737 (0.668 to 0.802) | 0.714 (0.631 to 0.788) | 2.52E-05 |

**Supplementary Table 6**. Common probes (n=141) across the 3 datasets (paired samples, SGBCC blood specimens, and SGBCC saliva specimens). Chr: Chromosome.

|  | **GRCh37/hg19** | | | |  | **Paired samples** | | **SGBCC blood specimens** | | **SGBCC saliva specimens** | |
| --- | --- | --- | --- | --- | --- | --- | --- | --- | --- | --- | --- |
| **Probes** | **Chr** | **Start** | **End** | **Strand** | **Symbol** | **Coefficients from *limma*** | **Uncorrected p-value** | **Coefficients from *limma*** | **Uncorrected p-value** | **Coefficients from *limma*** | **Uncorrected p-value** |
| cg00303773 | chr17 | 17752575 | 17752576 | - | *TOM1L2;TOM1L2;TOM1L2;TOM1L2;TOM1L2;TOM1L2* | -0.0362 | 3.86E-23 | -0.0290 | 1.14E-58 | -0.0243 | 2.45E-08 |
| cg08173263 | chr19 | 14276911 | 14276912 | - | *LPHN1;LPHN1* | 0.1307 | 1.18E-20 | 0.0718 | 4.10E-39 | 0.0874 | 6.28E-12 |
| cg26550214 | chr5 | 34609493 | 34609494 | + |  | -0.1090 | 2.33E-20 | -0.0577 | 7.73E-34 | -0.0632 | 1.84E-08 |
| cg11031221 | chr17 | 70417517 | 70417518 | + | *LINC00673* | -0.0766 | 1.47E-19 | -0.0572 | 4.80E-76 | -0.0473 | 1.30E-09 |
| cg15261712 | chr7 | 92238248 | 92238249 | + | *CDK6;CDK6* | 0.0692 | 5.48E-18 | 0.0408 | 1.57E-20 | 0.0730 | 3.80E-12 |
| cg01641754 | chr5 | 98355983 | 98355984 | - |  | -0.0590 | 3.56E-17 | -0.0414 | 1.95E-39 | -0.0386 | 7.77E-08 |
| cg15499265 | chr9 | 117157330 | 117157331 | - | *AKNA* | 0.0726 | 3.79E-16 | 0.0525 | 1.13E-34 | 0.0538 | 1.39E-08 |
| cg02485044 | chr13 | 50234343 | 50234344 | - |  | 0.0862 | 5.79E-16 | 0.0601 | 7.23E-40 | 0.0739 | 8.47E-13 |
| cg14792781 | chr2 | 10116243 | 10116244 | + | *GRHL1* | -0.0477 | 1.52E-15 | -0.0435 | 2.07E-61 | -0.0409 | 1.45E-09 |
| cg20396110 | chr15 | 74919916 | 74919917 | + | *CLK3;CLK3* | 0.0438 | 1.82E-15 | 0.0255 | 2.16E-29 | 0.0386 | 1.76E-12 |
| cg20507228 | chr15 | 91460071 | 91460072 | - | *MAN2A2* | 0.1152 | 2.58E-15 | 0.0653 | 1.06E-34 | 0.1011 | 1.79E-13 |
| cg06688763 | chr7 | 92238207 | 92238208 | + | *CDK6;CDK6* | 0.1160 | 2.80E-15 | 0.0784 | 1.39E-26 | 0.0737 | 8.41E-08 |
| cg07573872 | chr19 | 1126342 | 1126343 | - | *SBNO2;SBNO2* | -0.0768 | 3.42E-15 | -0.0563 | 1.81E-46 | -0.0690 | 1.58E-11 |
| cg01296603 | chr1 | 53751741 | 53751742 | + | *LRP8;LRP8;LRP8;LRP8* | 0.0715 | 4.69E-15 | 0.0610 | 3.33E-46 | 0.0537 | 4.90E-11 |
| cg25242306 | chr13 | 74667131 | 74667132 | - | *KLF12* | 0.0292 | 5.80E-15 | 0.0197 | 2.62E-31 | 0.0264 | 7.32E-08 |
| cg06946797 | chr16 | 11422409 | 11422410 | + |  | -0.0276 | 6.11E-15 | -0.0181 | 1.07E-26 | -0.0292 | 3.74E-09 |
| cg09618291 | chr2 | 149411300 | 149411301 | + | *EPC2* | 0.0424 | 6.52E-15 | 0.0247 | 5.62E-29 | 0.0301 | 2.05E-08 |
| cg26709300 | chr16 | 30106682 | 30106683 | - | *YPEL3;YPEL3* | 0.0689 | 7.68E-15 | 0.0554 | 8.88E-43 | 0.0620 | 1.17E-11 |
| cg14634473 | chr10 | 23105437 | 23105438 | - |  | -0.0694 | 2.02E-14 | -0.0539 | 8.95E-41 | -0.0557 | 1.60E-08 |
| cg21668652 | chr17 | 55749538 | 55749539 | - | *MSI2* | 0.0554 | 2.60E-14 | 0.0537 | 1.87E-41 | 0.0510 | 6.33E-09 |
| cg19078289 | chr3 | 152017293 | 152017294 | - | *MBNL1;MBNL1;MBNL1;MBNL1;MBNL1;MBNL1;MBNL1;MBNL1;MBNL1;MBNL1;MBNL1;MBNL1* | 0.0447 | 2.72E-14 | 0.0390 | 5.53E-40 | 0.0365 | 6.89E-08 |
| cg09187087 | chr11 | 45203304 | 45203305 | + | *PRDM11;PRDM11;PRDM11;PRDM11;PRDM11;PRDM11* | 0.0535 | 2.78E-14 | 0.0260 | 4.52E-16 | 0.0415 | 4.91E-08 |
| cg08709672 | chr1 | 206224334 | 206224335 | - | *AVPR1B;AVPR1B* | 0.0314 | 3.72E-14 | 0.0163 | 4.55E-16 | 0.0231 | 2.18E-08 |
| cg09225861 | chr11 | 65069680 | 65069681 | + |  | 0.0748 | 1.09E-13 | 0.0615 | 1.73E-28 | 0.0750 | 1.49E-09 |
| cg26377281 | chr12 | 4403352 | 4403353 | + | *CCND2* | 0.0283 | 1.19E-13 | 0.0167 | 6.08E-18 | 0.0307 | 1.29E-10 |
| cg05333719 | chr15 | 74921459 | 74921460 | - | *CLK3;CLK3* | 0.0338 | 1.61E-13 | 0.0207 | 1.33E-18 | 0.0301 | 1.17E-08 |
| cg15251748 | chr7 | 28993464 | 28993465 | + | *TRIL* | -0.0600 | 1.72E-13 | -0.0453 | 6.39E-35 | -0.0602 | 1.10E-08 |
| cg13702222 | chr3 | 152017240 | 152017241 | - | *MBNL1;MBNL1;MBNL1;MBNL1;MBNL1;MBNL1;MBNL1;MBNL1;MBNL1;MBNL1;MBNL1;MBNL1* | 0.0703 | 2.71E-13 | 0.0520 | 5.20E-37 | 0.0591 | 5.52E-09 |
| cg09022230 | chr7 | 5457225 | 5457226 | - | *TNRC18* | 0.0700 | 3.49E-13 | 0.0536 | 2.97E-33 | 0.0597 | 1.92E-08 |
| cg21478425 | chr22 | 39019601 | 39019602 | - | *FAM227A;FAM227A* | -0.0306 | 4.06E-13 | -0.0254 | 2.43E-44 | -0.0249 | 7.35E-08 |
| cg07413467 | chr7 | 92238086 | 92238087 | - | *CDK6;CDK6* | 0.0576 | 4.90E-13 | 0.0292 | 7.28E-12 | 0.0572 | 2.75E-10 |
| cg00591515 | chr19 | 54971526 | 54971527 | - | *LENG8* | 0.0532 | 6.33E-13 | 0.0458 | 3.56E-34 | 0.0463 | 6.95E-09 |
| cg17694094 | chr3 | 18484672 | 18484673 | - |  | 0.0812 | 1.08E-12 | 0.0591 | 7.59E-31 | 0.0742 | 3.69E-08 |
| cg14100654 | chr22 | 39146450 | 39146451 | + | *SUN2;SUN2;SUN2* | 0.0397 | 1.08E-12 | 0.0199 | 9.17E-18 | 0.0348 | 6.01E-08 |
| cg19532728 | chr11 | 65129859 | 65129860 | + |  | 0.0380 | 1.21E-12 | 0.0203 | 2.70E-14 | 0.0448 | 7.51E-10 |
| cg17103217 | chr6 | 33245721 | 33245722 | + | *B3GALT4* | 0.0251 | 1.45E-12 | 0.0182 | 3.88E-17 | 0.0320 | 6.79E-08 |
| cg08899667 | chr6 | 31761055 | 31761056 | - | *VARS* | 0.0532 | 1.46E-12 | 0.0320 | 3.18E-24 | 0.0547 | 9.28E-12 |
| cg18265326 | chr16 | 65635738 | 65635739 | - |  | 0.0874 | 1.55E-12 | 0.0786 | 1.99E-43 | 0.0936 | 5.74E-11 |
| cg20952257 | chr5 | 171074407 | 171074408 | + |  | -0.0424 | 1.76E-12 | -0.0376 | 1.60E-49 | -0.0319 | 2.13E-08 |
| cg22878489 | chr6 | 33245701 | 33245702 | + | *B3GALT4* | 0.0384 | 1.88E-12 | 0.0339 | 1.21E-24 | 0.0541 | 1.04E-08 |
| cg27115863 | chr22 | 37921640 | 37921641 | - |  | -0.0489 | 2.48E-12 | -0.0519 | 2.63E-49 | -0.0591 | 1.88E-10 |
| cg01526748 | chr3 | 191930926 | 191930927 | - | *FGF12;FGF12* | 0.0594 | 2.58E-12 | 0.0458 | 6.38E-18 | 0.0888 | 2.12E-08 |
| cg27330053 | chr2 | 153057298 | 153057299 | - |  | -0.0560 | 2.70E-12 | -0.0591 | 4.23E-45 | -0.0518 | 4.54E-08 |
| cg09225336 | chr16 | 65635750 | 65635751 | + |  | 0.0881 | 2.78E-12 | 0.0774 | 1.03E-40 | 0.1015 | 3.70E-12 |
| cg02194129 | chr14 | 104171313 | 104171314 | + | *XRCC3;XRCC3;XRCC3* | 0.0456 | 3.06E-12 | 0.0311 | 7.01E-29 | 0.0411 | 2.07E-09 |
| cg11387709 | chr16 | 65636127 | 65636128 | - |  | 0.1006 | 3.18E-12 | 0.0719 | 1.88E-29 | 0.0979 | 6.74E-08 |
| cg09800781 | chr10 | 104253015 | 104253016 | + | *ACTR1A* | 0.0461 | 3.76E-12 | 0.0235 | 7.44E-21 | 0.0364 | 3.11E-09 |
| cg21842033 | chr9 | 132752919 | 132752920 | + | *FNBP1* | 0.0291 | 4.44E-12 | 0.0184 | 2.41E-18 | 0.0329 | 5.16E-10 |
| cg17344906 | chr19 | 13202507 | 13202508 | - | *NFIX* | 0.0485 | 5.40E-12 | 0.0384 | 4.93E-32 | 0.0562 | 1.10E-09 |
| cg24899334 | chr3 | 167803450 | 167803451 | + | *GOLIM4;GOLIM4* | -0.0669 | 5.41E-12 | -0.0619 | 9.06E-40 | -0.0773 | 3.55E-09 |
| cg16595241 | chr3 | 100354385 | 100354386 | + | *ADGRG7;ADGRG7;ADGRG7* | 0.0581 | 5.86E-12 | 0.0337 | 3.82E-19 | 0.0491 | 9.39E-09 |
| cg12798040 | chr14 | 104171840 | 104171841 | - | *XRCC3;XRCC3;XRCC3* | 0.0666 | 6.85E-12 | 0.0631 | 1.90E-51 | 0.0619 | 2.10E-11 |
| cg27340201 | chr12 | 66342303 | 66342304 | - | *HMGA2;HMGA2* | 0.1045 | 7.08E-12 | 0.0678 | 2.59E-34 | 0.0740 | 6.12E-09 |
| cg03561416 | chr6 | 157254584 | 157254585 | + | *ARID1B;ARID1B;ARID1B* | 0.0361 | 8.52E-12 | 0.0323 | 7.33E-33 | 0.0447 | 4.57E-12 |
| cg06991118 | chr18 | 9117348 | 9117349 | + | *NDUFV2* | -0.0586 | 8.64E-12 | -0.0514 | 2.02E-44 | -0.0710 | 1.07E-10 |
| cg23605961 | chr7 | 751331 | 751332 | - | *PRKAR1B;PRKAR1B;PRKAR1B;PRKAR1B;PRKAR1B;PRKAR1B* | 0.0681 | 1.00E-11 | 0.0329 | 5.12E-14 | 0.0768 | 5.44E-11 |
| cg01655226 | chr17 | 16955974 | 16955975 | - | *MPRIP;MPRIP* | 0.0456 | 1.07E-11 | 0.0383 | 7.73E-15 | 0.0606 | 3.15E-08 |
| cg07687574 | chr17 | 8844581 | 8844582 | + | *PIK3R5;PIK3R5;PIK3R5;PIK3R5;PIK3R5* | -0.0253 | 1.22E-11 | -0.0249 | 2.71E-48 | -0.0257 | 3.13E-08 |
| cg18745690 | chr7 | 99754227 | 99754228 | + | *C7orf43;C7orf43;MIR4658* | 0.0268 | 1.52E-11 | 0.0233 | 1.56E-28 | 0.0357 | 1.31E-10 |
| cg05914119 | chr15 | 74877163 | 74877164 | + | *ARID3B;ARID3B* | 0.0631 | 1.72E-11 | 0.0278 | 2.05E-12 | 0.0502 | 2.18E-08 |
| cg10207189 | chr13 | 51185590 | 51185591 | - |  | 0.0219 | 2.09E-11 | 0.0101 | 2.38E-09 | 0.0281 | 8.86E-09 |
| cg12585732 | chr10 | 80920577 | 80920578 | + | *ZMIZ1* | 0.0411 | 2.37E-11 | 0.0223 | 2.23E-14 | 0.0454 | 3.80E-10 |
| cg08115371 | chr11 | 79115706 | 79115707 | + | *ODZ4* | 0.0560 | 2.61E-11 | 0.0390 | 2.09E-19 | 0.0736 | 1.00E-10 |
| cg04460609 | chr4 | 16532808 | 16532809 | - | *LDB2;LDB2* | 0.0493 | 2.99E-11 | 0.0493 | 6.40E-41 | 0.0570 | 2.32E-09 |
| cg17038235 | chr5 | 172370524 | 172370525 | + | *ERGIC1* | 0.0494 | 3.03E-11 | 0.0260 | 8.82E-20 | 0.0462 | 1.70E-09 |
| cg01478827 | chr1 | 21558579 | 21558580 | - | *ECE1;ECE1;ECE1;ECE1* | 0.0477 | 3.60E-11 | 0.0330 | 2.08E-19 | 0.0528 | 7.56E-09 |
| cg18537183 | chr14 | 104170490 | 104170491 | + | *XRCC3;XRCC3;XRCC3* | 0.0389 | 4.26E-11 | 0.0261 | 9.98E-18 | 0.0483 | 9.69E-11 |
| cg02016405 | chr2 | 65297792 | 65297793 | + | *CEP68* | -0.0343 | 4.59E-11 | -0.0347 | 1.36E-44 | -0.0356 | 6.96E-09 |
| cg17552710 | chr5 | 139062999 | 139063000 | - |  | 0.0362 | 5.59E-11 | 0.0352 | 1.66E-29 | 0.0432 | 1.32E-11 |
| cg01874808 | chr5 | 150074466 | 150074467 | - | *RBM22* | 0.0633 | 6.60E-11 | 0.0338 | 7.64E-24 | 0.0526 | 2.71E-10 |
| cg19420069 | chr16 | 65635834 | 65635835 | + |  | 0.0983 | 7.07E-11 | 0.0900 | 2.70E-41 | 0.1088 | 1.11E-10 |
| cg08843623 | chr16 | 85627648 | 85627649 | + |  | 0.0455 | 8.02E-11 | 0.0265 | 1.05E-16 | 0.0507 | 6.12E-10 |
| cg14088282 | chr1 | 222003220 | 222003221 | - |  | -0.1083 | 8.06E-11 | -0.1158 | 1.75E-58 | -0.1027 | 4.58E-08 |
| cg13379236 | chr4 | 110833808 | 110833809 | - | *EGF* | 0.0476 | 8.88E-11 | 0.0318 | 1.35E-17 | 0.0540 | 6.83E-09 |
| cg10351852 | chr3 | 45706990 | 45706991 | + | *LIMD1* | 0.0321 | 9.96E-11 | 0.0223 | 2.60E-20 | 0.0332 | 4.20E-09 |
| cg08913955 | chr15 | 75276450 | 75276451 | + |  | -0.0371 | 1.11E-10 | -0.0361 | 8.59E-58 | -0.0324 | 5.68E-09 |
| cg21329416 | chr1 | 27045929 | 27045930 | + | *ARID1A;ARID1A* | 0.0352 | 1.21E-10 | 0.0261 | 2.96E-19 | 0.0394 | 1.54E-09 |
| cg22867359 | chr16 | 17570564 | 17570565 | - |  | 0.0241 | 1.21E-10 | 0.0109 | 1.13E-13 | 0.0206 | 5.89E-08 |
| cg18152558 | chr4 | 75956216 | 75956217 | + | *PARM1* | 0.0743 | 1.28E-10 | 0.0486 | 2.36E-23 | 0.0858 | 3.29E-09 |
| cg27646484 | chr16 | 31210736 | 31210737 | + |  | 0.0376 | 1.39E-10 | 0.0211 | 6.41E-19 | 0.0407 | 1.01E-11 |
| cg01349024 | chr1 | 26102026 | 26102027 | + | *MAN1C1;MAN1C1* | 0.0281 | 2.09E-10 | 0.0125 | 4.51E-14 | 0.0252 | 6.19E-08 |
| cg22169301 | chr19 | 40780424 | 40780425 | + | *AKT2;AKT2;AKT2* | 0.0334 | 2.41E-10 | 0.0261 | 4.55E-23 | 0.0357 | 4.31E-08 |
| cg25739436 | chr3 | 42135664 | 42135665 | - | *TRAK1;TRAK1* | 0.0389 | 2.84E-10 | 0.0152 | 2.04E-08 | 0.0363 | 3.28E-08 |
| cg23580000 | chr16 | 50322156 | 50322157 | + | *ADCY7* | 0.0351 | 3.38E-10 | 0.0371 | 6.88E-31 | 0.0470 | 3.42E-10 |
| cg16596317 | chr1 | 85755348 | 85755349 | + |  | -0.0650 | 5.59E-10 | -0.0748 | 1.24E-38 | -0.0675 | 8.16E-08 |
| cg13065415 | chr17 | 3858758 | 3858759 | - | *ATP2A3;ATP2A3;ATP2A3;ATP2A3;ATP2A3;ATP2A3;ATP2A3* | 0.0474 | 5.78E-10 | 0.0257 | 4.31E-16 | 0.0511 | 9.61E-10 |
| cg20731257 | chr2 | 87883497 | 87883498 | + |  | 0.0307 | 6.02E-10 | 0.0173 | 2.72E-12 | 0.0338 | 5.07E-09 |
| cg05645538 | chr8 | 56858948 | 56858949 | - | *LYN;LYN* | 0.0504 | 6.13E-10 | 0.0324 | 3.67E-19 | 0.0495 | 2.94E-10 |
| cg03127244 | chr6 | 33245638 | 33245639 | + | *B3GALT4* | 0.0357 | 8.90E-10 | 0.0372 | 2.06E-25 | 0.0534 | 8.21E-09 |
| cg06034584 | chr9 | 32566125 | 32566126 | + | *TOPORS-AS1;TOPORS-AS1;NDUFB6;NDUFB6;NDUFB6* | 0.0394 | 8.98E-10 | 0.0241 | 1.90E-12 | 0.0494 | 1.33E-08 |
| cg03984067 | chr17 | 55348624 | 55348625 | + | *MSI2;MSI2* | 0.0253 | 9.63E-10 | 0.0119 | 2.54E-15 | 0.0217 | 4.75E-08 |
| cg23486701 | chr2 | 54789491 | 54789492 | - | *SPTBN1;SPTBN1* | 0.0409 | 9.82E-10 | 0.0349 | 5.24E-14 | 0.0617 | 6.02E-08 |
| cg18647570 | chr17 | 41594616 | 41594617 | + | *DHX8* | 0.0441 | 1.07E-09 | 0.0242 | 2.19E-12 | 0.0505 | 4.70E-08 |
| cg27403957 | chr19 | 40786376 | 40786377 | + | *AKT2;AKT2;AKT2* | 0.0342 | 1.13E-09 | 0.0249 | 5.99E-27 | 0.0328 | 3.87E-08 |
| cg03337382 | chr10 | 125960385 | 125960386 | + |  | -0.0447 | 1.18E-09 | -0.0514 | 4.55E-36 | -0.0455 | 1.54E-08 |
| cg13707760 | chr9 | 95986383 | 95986384 | - | *WNK2* | 0.0364 | 1.31E-09 | 0.0181 | 1.01E-08 | 0.0426 | 7.96E-08 |
| cg02879453 | chr16 | 50321818 | 50321819 | - | *ADCY7* | 0.0430 | 1.56E-09 | 0.0447 | 1.34E-34 | 0.0544 | 2.33E-09 |
| cg05926358 | chr9 | 96264047 | 96264048 | + | *FAM120A;FAM120A;FAM120A;FAM120A* | 0.0519 | 1.84E-09 | 0.0380 | 4.68E-29 | 0.0421 | 1.61E-09 |
| cg26432350 | chr3 | 47040357 | 47040358 | - | *NBEAL2* | 0.0368 | 2.10E-09 | 0.0321 | 1.15E-45 | 0.0482 | 1.51E-13 |
| cg05903330 | chr22 | 39542136 | 39542137 | + | *CBX7* | 0.0224 | 2.18E-09 | 0.0097 | 1.31E-14 | 0.0170 | 2.74E-09 |
| cg04492438 | chr12 | 57997387 | 57997388 | + | *DTX3;DTX3;DTX3* | 0.0310 | 2.18E-09 | 0.0143 | 7.39E-13 | 0.0339 | 3.61E-08 |
| cg17277833 | chr2 | 43558544 | 43558545 | + | *THADA;THADA;THADA* | 0.0554 | 2.76E-09 | 0.0546 | 2.97E-39 | 0.0599 | 3.43E-08 |
| cg12029753 | chr3 | 55650172 | 55650173 | + | *ERC2* | 0.0361 | 2.87E-09 | 0.0234 | 5.47E-21 | 0.0441 | 2.69E-10 |
| cg03398055 | chr17 | 55404210 | 55404211 | + | *MSI2;MSI2* | 0.0345 | 3.15E-09 | 0.0233 | 2.01E-20 | 0.0335 | 5.06E-08 |
| cg07330123 | chr16 | 3005479 | 3005480 | - |  | 0.0278 | 3.23E-09 | 0.0220 | 2.79E-14 | 0.0364 | 1.61E-08 |
| cg06581459 | chr19 | 6577668 | 6577669 | + |  | -0.0651 | 3.26E-09 | -0.0779 | 1.90E-48 | -0.0758 | 8.32E-08 |
| cg20460227 | chr2 | 120452632 | 120452633 | + |  | 0.0353 | 3.69E-09 | 0.0168 | 1.16E-10 | 0.0410 | 2.20E-09 |
| cg09874683 | chr9 | 129925853 | 129925854 | + | *RALGPS1;RALGPS1;RALGPS1;RALGPS1* | -0.0497 | 3.81E-09 | -0.0517 | 2.21E-36 | -0.0780 | 5.13E-10 |
| cg09435170 | chr4 | 184256627 | 184256628 | + |  | -0.0761 | 3.95E-09 | -0.0897 | 6.84E-50 | -0.0873 | 2.27E-09 |
| cg09783609 | chr11 | 36322367 | 36322368 | - | *PRR5L* | 0.0486 | 4.04E-09 | 0.0213 | 6.31E-10 | 0.0427 | 7.15E-08 |
| cg26642703 | chr3 | 196389926 | 196389927 | - |  | 0.0494 | 4.10E-09 | 0.0388 | 5.85E-22 | 0.0669 | 2.25E-11 |
| cg05779272 | chr9 | 33168018 | 33168019 | + | *B4GALT1* | 0.0204 | 5.15E-09 | 0.0155 | 5.01E-22 | 0.0215 | 2.44E-08 |
| cg08991437 | chr3 | 14963073 | 14963074 | + | *FGD5* | 0.0338 | 5.41E-09 | 0.0252 | 3.38E-17 | 0.0427 | 1.46E-10 |
| cg06116549 | chr20 | 45963043 | 45963044 | - | *ZMYND8;ZMYND8;ZMYND8;ZMYND8;ZMYND8;ZMYND8;ZMYND8;ZMYND8;ZMYND8;ZMYND8;ZMYND8;ZMYND8;ZMYND8;ZMYND8;ZMYND8;ZMYND8;ZMYND8;ZMYND8* | 0.0495 | 5.67E-09 | 0.0371 | 1.57E-24 | 0.0572 | 3.76E-09 |
| cg09276451 | chr16 | 4421603 | 4421604 | - | *VASN;CORO7* | 0.0360 | 6.40E-09 | 0.0210 | 1.28E-12 | 0.0360 | 1.41E-08 |
| cg07172800 | chr11 | 457742 | 457743 | - | *PTDSS2* | 0.0316 | 7.63E-09 | 0.0204 | 9.17E-16 | 0.0370 | 5.19E-09 |
| cg10466675 | chr1 | 212214107 | 212214108 | + | *DTL;DTL;DTL* | -0.0404 | 8.03E-09 | -0.0450 | 8.33E-33 | -0.0503 | 7.45E-08 |
| cg17472649 | chr11 | 35835797 | 35835798 | + |  | 0.0546 | 8.29E-09 | 0.0224 | 3.46E-11 | 0.0473 | 2.54E-08 |
| cg20231986 | chr19 | 15428689 | 15428690 | + |  | 0.0228 | 9.35E-09 | 0.0094 | 3.65E-13 | 0.0155 | 7.01E-08 |
| cg20498634 | chr10 | 44506655 | 44506656 | + |  | -0.0258 | 9.65E-09 | -0.0169 | 1.26E-21 | -0.0291 | 5.69E-08 |
| cg24127061 | chr11 | 65839402 | 65839403 | + | *PACS1* | 0.0361 | 1.05E-08 | 0.0317 | 1.02E-24 | 0.0480 | 1.42E-08 |
| cg03052074 | chr13 | 41543655 | 41543656 | + | *ELF1;ELF1* | 0.0401 | 1.10E-08 | 0.0418 | 3.16E-29 | 0.0452 | 5.44E-08 |
| cg25984701 | chr13 | 52006860 | 52006861 | - | *INTS6;INTS6;INTS6* | 0.0423 | 1.16E-08 | 0.0296 | 7.54E-15 | 0.0612 | 2.41E-09 |
| cg16528611 | chr1 | 226256663 | 226256664 | + | *H3F3AP4;H3F3A* | 0.0380 | 1.27E-08 | 0.0310 | 2.15E-19 | 0.0484 | 7.53E-08 |
| cg03824899 | chr12 | 50031340 | 50031341 | - | *PRPF40B;PRPF40B* | 0.0294 | 1.27E-08 | 0.0123 | 2.32E-11 | 0.0275 | 8.84E-09 |
| cg10541774 | chr2 | 137068792 | 137068793 | - |  | 0.0391 | 1.61E-08 | 0.0483 | 1.69E-40 | 0.0507 | 4.52E-08 |
| cg23667692 | chr19 | 33615760 | 33615761 | + | *GPATCH1* | 0.0296 | 1.66E-08 | 0.0150 | 2.02E-12 | 0.0300 | 2.46E-08 |
| cg00073565 | chr8 | 106462708 | 106462709 | + | *ZFPM2* | 0.0563 | 1.74E-08 | 0.0321 | 5.32E-16 | 0.0519 | 1.97E-08 |
| cg03295554 | chr11 | 128395450 | 128395451 | + | *ETS1* | -0.0780 | 1.80E-08 | -0.0768 | 4.10E-40 | -0.0836 | 4.68E-09 |
| cg16353628 | chr2 | 85239916 | 85239917 | + | *KCMF1* | 0.0447 | 2.29E-08 | 0.0380 | 7.80E-23 | 0.0568 | 2.74E-09 |
| cg06225648 | chr9 | 109976075 | 109976076 | - |  | -0.0233 | 2.82E-08 | -0.0218 | 1.50E-38 | -0.0309 | 3.98E-09 |
| cg26599989 | chr11 | 1297087 | 1297088 | - | *TOLLIP* | 0.0325 | 2.89E-08 | 0.0341 | 1.41E-27 | 0.0418 | 4.72E-09 |
| cg04622454 | chr9 | 140349128 | 140349129 | - | *NELF;NELF;NELF;NELF* | 0.0300 | 3.41E-08 | 0.0129 | 5.87E-10 | 0.0293 | 1.30E-08 |
| cg16736826 | chr1 | 41951512 | 41951513 | - | *EDN2* | -0.0205 | 3.44E-08 | -0.0193 | 1.78E-30 | -0.0243 | 2.90E-08 |
| cg02491871 | chr12 | 11911896 | 11911897 | + | *ETV6* | 0.0354 | 3.58E-08 | 0.0231 | 2.71E-16 | 0.0446 | 1.22E-09 |
| cg21618521 | chr6 | 33245770 | 33245771 | - | *B3GALT4* | 0.0214 | 3.90E-08 | 0.0190 | 4.20E-16 | 0.0348 | 2.16E-08 |
| cg03326609 | chr4 | 124165382 | 124165383 | + | *SPATA5* | 0.0506 | 3.97E-08 | 0.0288 | 1.74E-14 | 0.0481 | 1.43E-09 |
| cg09082427 | chr9 | 140349184 | 140349185 | + | *NSMF;NSMF;NSMF;NSMF;NSMF* | 0.0351 | 4.84E-08 | 0.0239 | 4.12E-21 | 0.0521 | 9.10E-10 |
| cg03315921 | chr22 | 18243630 | 18243631 | - | *BID;BID;BID;BID;BID;BID;BID* | 0.0471 | 5.00E-08 | 0.0550 | 2.94E-35 | 0.0578 | 3.58E-08 |
| cg01756827 | chr20 | 47923790 | 47923791 | + |  | 0.0351 | 5.54E-08 | 0.0294 | 1.95E-14 | 0.0517 | 7.90E-08 |
| cg01751802 | chr19 | 11309639 | 11309640 | - | *KANK2* | 0.0480 | 6.10E-08 | 0.0314 | 5.74E-15 | 0.0567 | 7.63E-09 |

**Supplementary Table 7.** Promoters (n=11) that remained significant after multiple testing correction across all three datasets (paired samples, SGBCC blood specimens, and SGBCC saliva specimens).

|  | **GRCh37/hg19** | | | |  | **Paired samples** | | **SGBCC blood specimens** | | **SGBCC saliva specimens** | |
| --- | --- | --- | --- | --- | --- | --- | --- | --- | --- | --- | --- |
| **Promoter** | **Chromosome** | **Start** | **End** | **Strand** | **Symbol** | **Coefficients from *limma*** | **Uncorrected p-value** | **Coefficients from *limma*** | **Uncorrected p-value** | **Coefficients from *limma*** | **Uncorrected p-value** |
| ENSG00000267610 | chr19 | 13181689 | 13183688 | - |  | 0.0177 | 2.21E-14 | 0.0055 | 5.23E-10 | 0.0126 | 4.19E-07 |
| ENSG00000260004 | chr16 | 65635102 | 65637101 | - |  | 0.0621 | 8.01E-13 | 0.0534 | 4.43E-42 | 0.0673 | 3.86E-11 |
| ENSG00000263847 | chr18 | 9115376 | 9117375 | - |  | -0.0586 | 1.01E-11 | -0.0514 | 2.10E-44 | -0.0710 | 1.11E-10 |
| ENSG00000256083 | chr12 | 66341760 | 66343759 | - |  | 0.0477 | 2.56E-11 | 0.0213 | 1.59E-27 | 0.0235 | 6.41E-07 |
| ENSG00000213859 | chr17 | 7253708 | 7255707 | + | *KCTD11* | 0.0247 | 4.87E-11 | 0.0189 | 3.89E-14 | 0.0291 | 2.06E-07 |
| ENSG00000207223 | chr15 | 64943587 | 64945586 | + |  | 0.0459 | 6.04E-11 | 0.0273 | 3.63E-22 | 0.0402 | 1.22E-07 |
| ENSG00000250616 | chr16 | 30106251 | 30108250 | + |  | 0.0220 | 8.98E-11 | 0.0182 | 1.61E-38 | 0.0188 | 2.32E-10 |
| ENSG00000262482 | chr16 | 3003778 | 3005777 | - |  | 0.0146 | 2.02E-10 | 0.0104 | 1.53E-09 | 0.0185 | 8.96E-07 |
| ENSG00000249526 | chr5 | 139062730 | 139064729 | + |  | 0.0187 | 4.50E-10 | 0.0177 | 4.21E-29 | 0.0239 | 4.66E-13 |
| ENSG00000250984 | chr5 | 29794545 | 29796544 | + |  | 0.0456 | 3.11E-09 | 0.0305 | 1.95E-09 | 0.0590 | 1.84E-07 |
| ENSG00000106948 | chr9 | 117156186 | 117158185 | - | *AKNA* | 0.0229 | 6.93E-07 | 0.0158 | 4.52E-20 | 0.0243 | 2.83E-07 |
