# Supplementary material for: Alterations to DNA methylation patterns induced by chemotherapy treatment are associated with negative impacts on the olfactory pathway
Source: Breast Cancer Res. 2023 Nov 6;25:136. doi: 10.1186/s13058-023-01730-4 (PMC10626732; doi:10.1186/s13058-023-01730-4)

**Additional file – Figure 1**. Manhattan plot of association results between chemotherapy and 525,100 DNA methylation in CpG probes in 1,808 breast cancer patients with blood specimens (1,066 with chemotherapy and 742 without chemotherapy). Presented here are associations that reached statistical significance at a Bonferroni threshold p-value (p<9.52e-8) with adjustments for batch, immune cell content (LUMP), cell types, age at breast cancer diagnosis, year of breast cancer diagnosis, and ethnicity (Additional file 7).


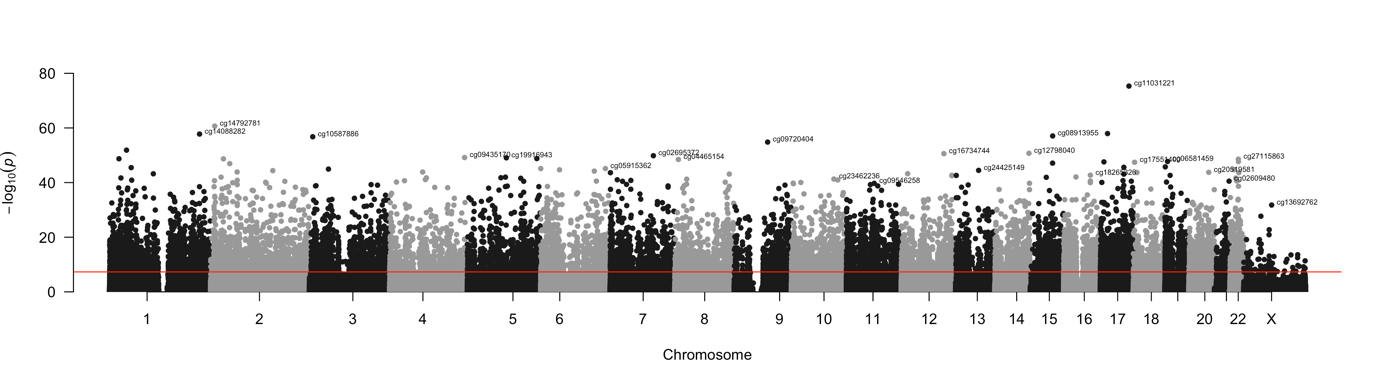


**Additional file – Figure 2**. Manhattan plot of association results between chemotherapy and 525,100 DNA methylation in CpG probes in 337 breast cancer patients with saliva specimens (207 with chemotherapy and 130 without chemotherapy). Presented here are associations that reached statistical significance at a Bonferroni threshold p-value (p<9.52e-8) with adjustments for batch, immune cell content (LUMP), age at breast cancer diagnosis, year of breast cancer diagnosis, and ethnicity (Additional file 8).

**
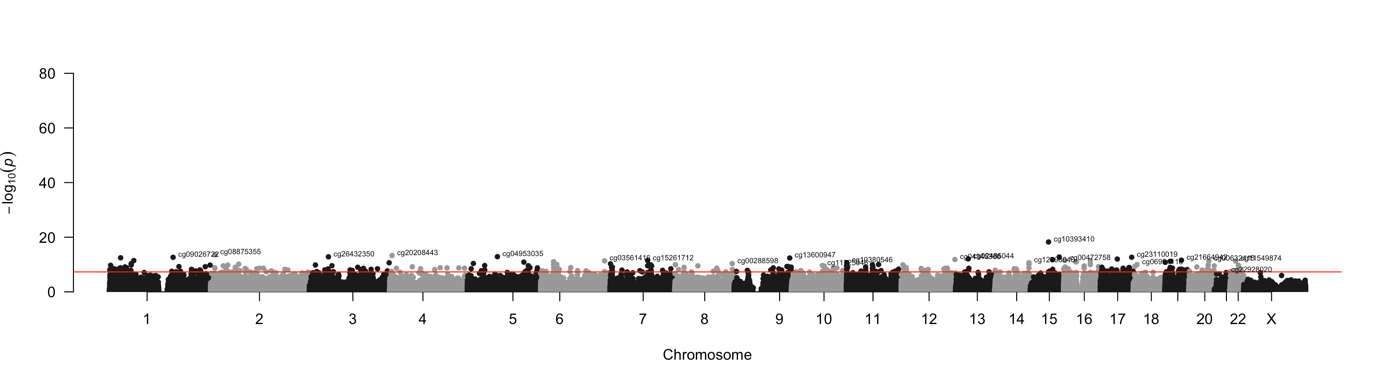
**

**Additional file – Figure 3**. Top 5 activated and 5 suppressed enriched Gene Ontology (GO) biological processes using associations from adjusted models (adjusted for batch, immune cell content (LUMP), cell type, age at diagnosis, year of diagnosis, and ethnicity), in blood samples of 1,808 breast cancer patients (1,066 with chemotherapy and 742 without chemotherapy).


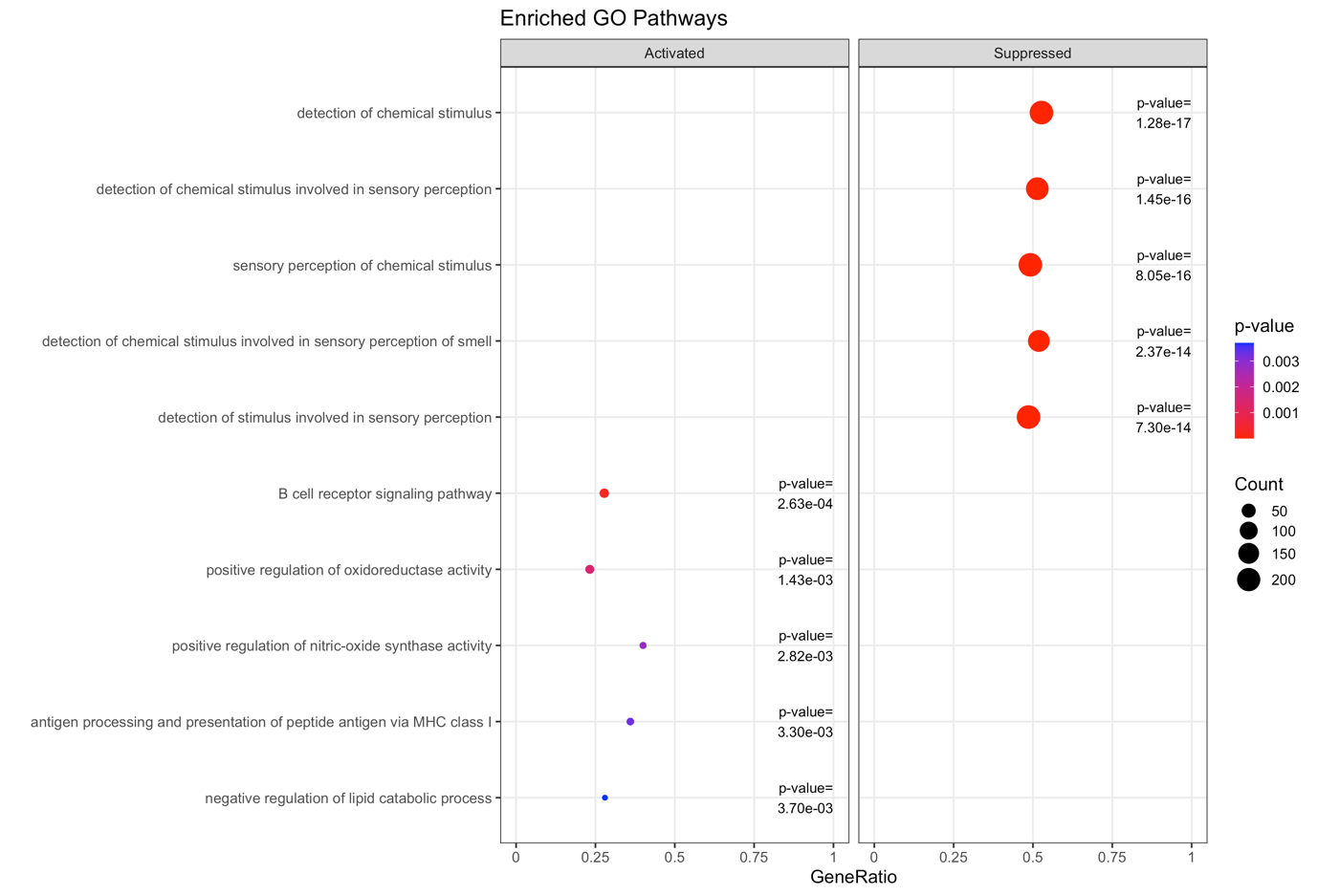


**Additional file – Figure 4**. Top 5 activated and 5 suppressed Gene Ontology (GO) biological processes using associations from adjusted models (adjusted for batch, immune cell content (LUMP), cell type, age at diagnosis, year of diagnosis, and ethnicity), in saliva samples of 337 breast cancer patients (207 with chemotherapy and 130 without chemotherapy).


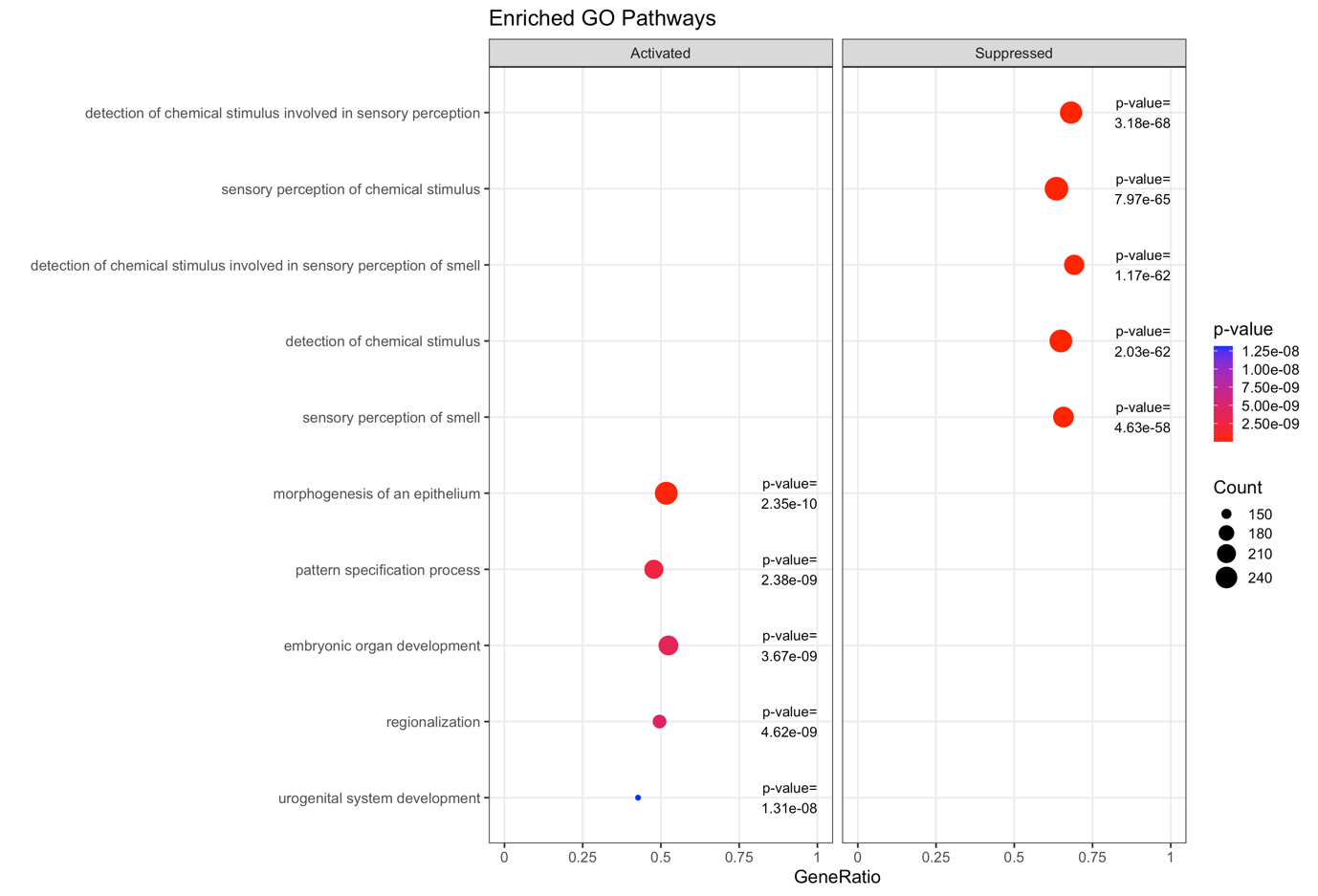


**Additional file – Figure 5**. Top 5 activated and 5 suppressed enriched KEGG pathways using associations from adjusted models (adjusted for batch, immune cell content (LUMP), cell type, age at diagnosis, year of diagnosis, and ethnicity), in blood samples of 1,808 breast cancer patients (1,066 with chemotherapy and 742 without chemotherapy).


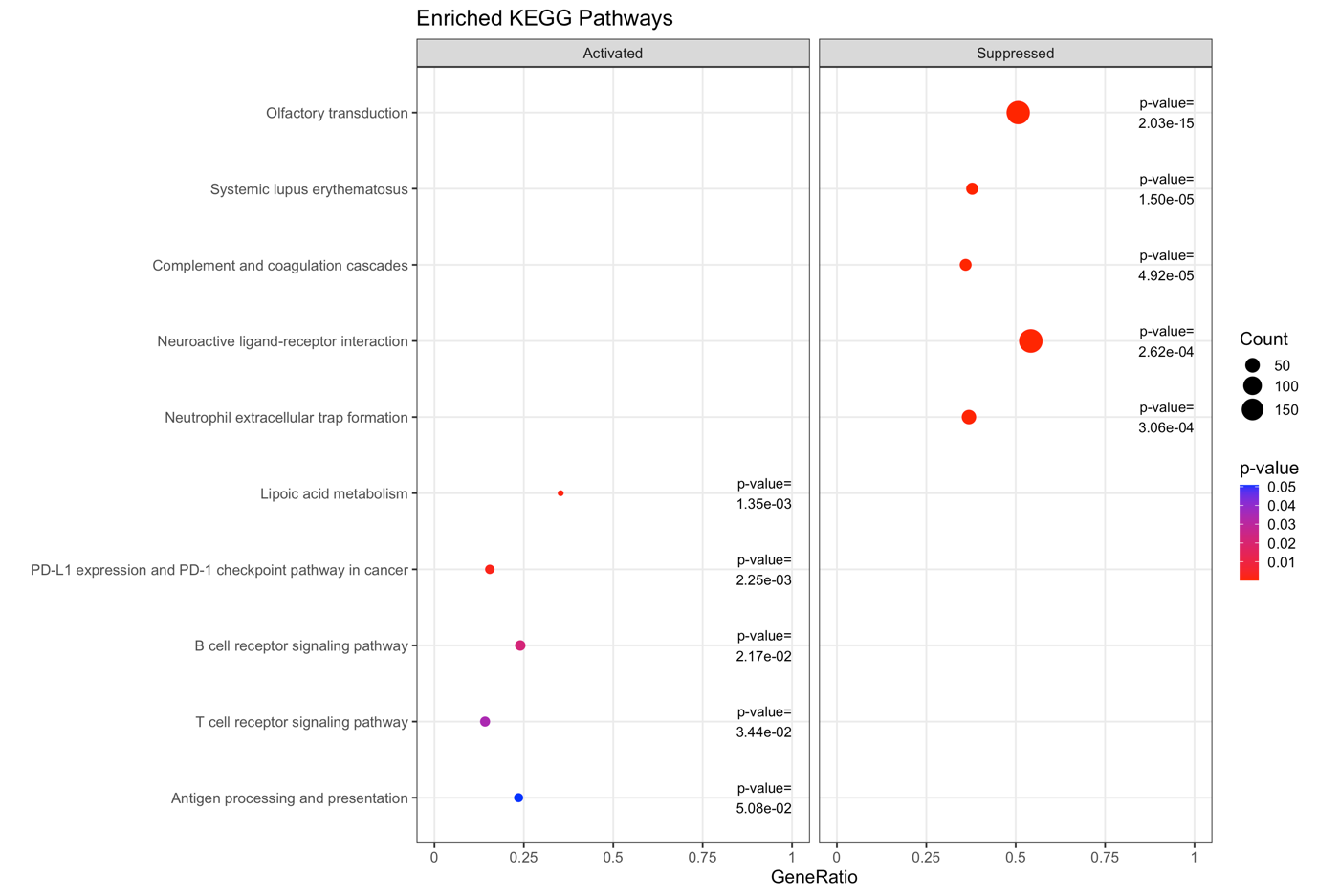


**Additional file – Figure 6**. Top 5 activated and 5 suppressed enriched KEGG pathways using associations from adjusted models (adjusted for batch, immune cell content (LUMP), age at diagnosis, year of diagnosis, and ethnicity), in saliva samples of 337 breast cancer patients (207 with chemotherapy and 130 without chemotherapy).


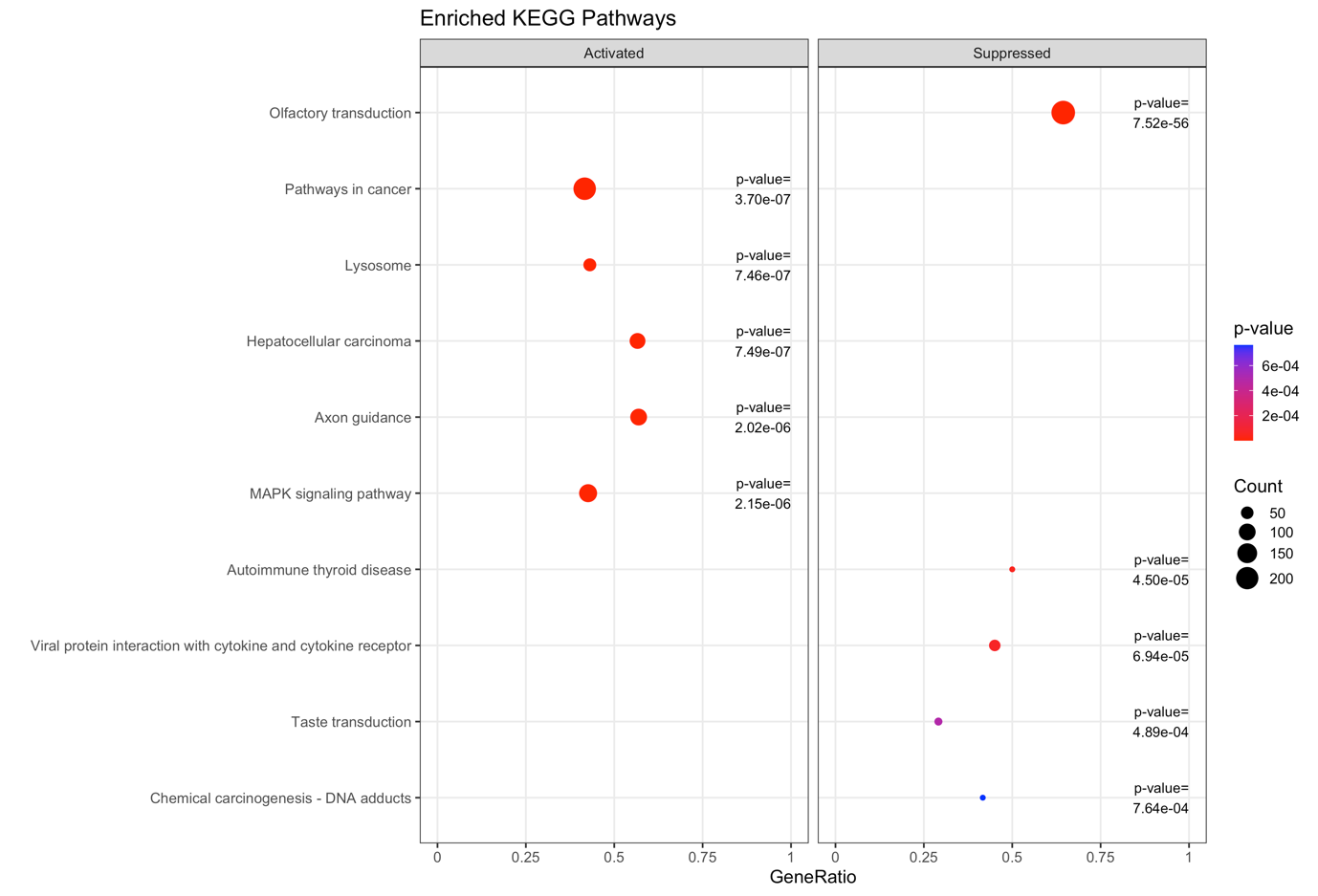


**Additional file – Figure 7**. Manhattan plot presenting association results between chemotherapy (samples obtained <0.5 year from chemotherapy start date vs. no chemotherapy) and 525,100 DNA methylation in CpG probes in breast cancer patients with blood specimens. Presented here are associations that reached statistical significance at a Bonferroni threshold p-value (p<9.52e-8) with adjustments for batch, immune cell content (LUMP), cell types, age at breast cancer diagnosis, and ethnicity (Additional file 13).


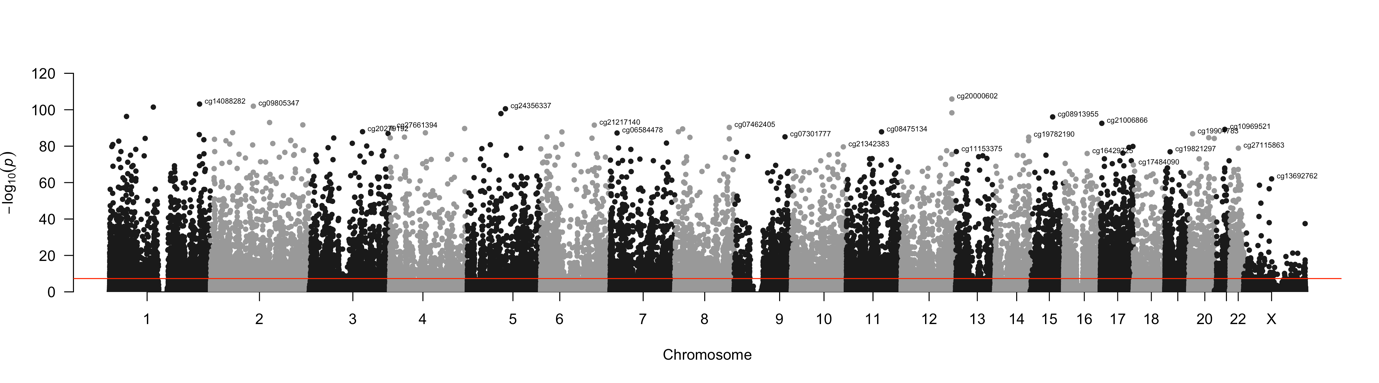


**Additional file – Figure 8**. Manhattan plot presenting association results between chemotherapy (samples obtained 0.5 to 2 years from chemotherapy start date vs. no chemotherapy) and 525,100 DNA methylation in CpG probes in breast cancer patients with blood specimens. Presented here are associations that reached statistical significance at a Bonferroni threshold p-value (p<9.52e-8) with adjustments for batch, immune cell content (LUMP), cell types, age at breast cancer diagnosis, and ethnicity (Additional file 13).


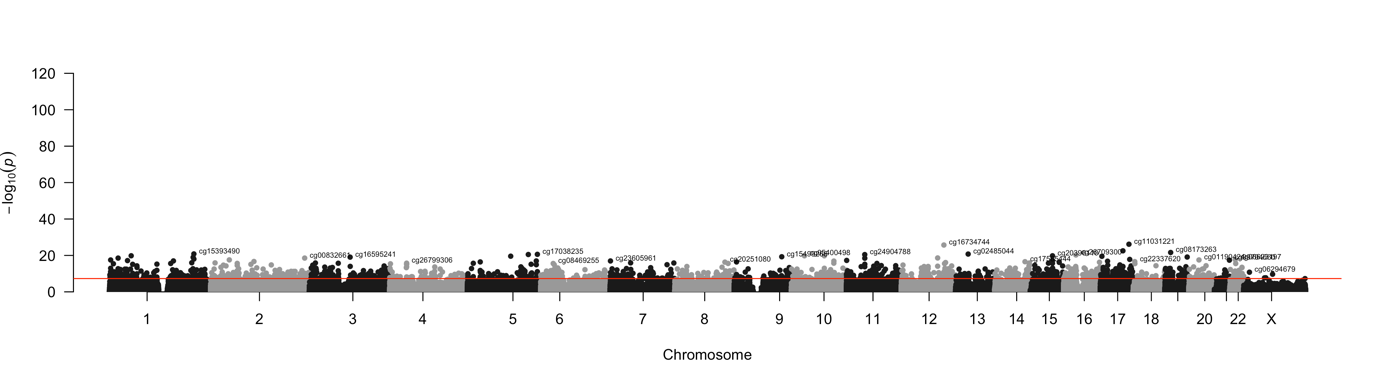


**Additional file – Figure 9**. Manhattan plot presenting association results between chemotherapy (Samples obtained >2 years from chemotherapy start date vs. no chemotherapy) and 525,100 DNA methylation in CpG probes in breast cancer patients with blood specimens. Presented here are associations that reached statistical significance at a Bonferroni threshold p-value (p<9.52e-8) with adjustments for batch, immune cell content (LUMP), cell types, age at breast cancer diagnosis, and ethnicity (Additional file 13).


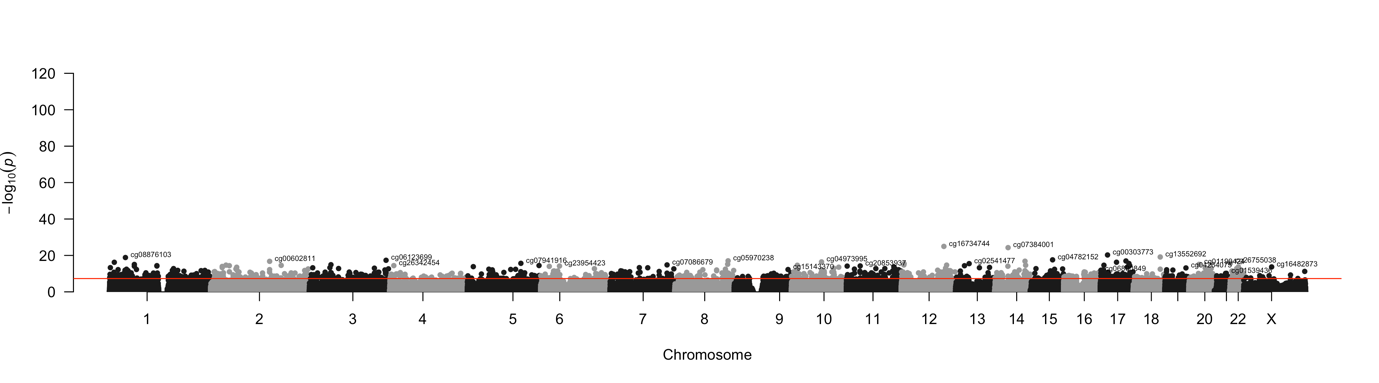


**Additional file – Figure 10**. Manhattan plot presenting association results between chemotherapy (samples obtained <0.5 year from chemotherapy start date vs. no chemotherapy) and 525,100 DNA methylation in CpG probes in breast cancer patients with saliva specimens. Presented here are associations that reached statistical significance at a Bonferroni threshold p-value (p<9.52e-8) with adjustments for batch, immune cell content (LUMP), age at breast cancer diagnosis, and ethnicity (Additional file 13).


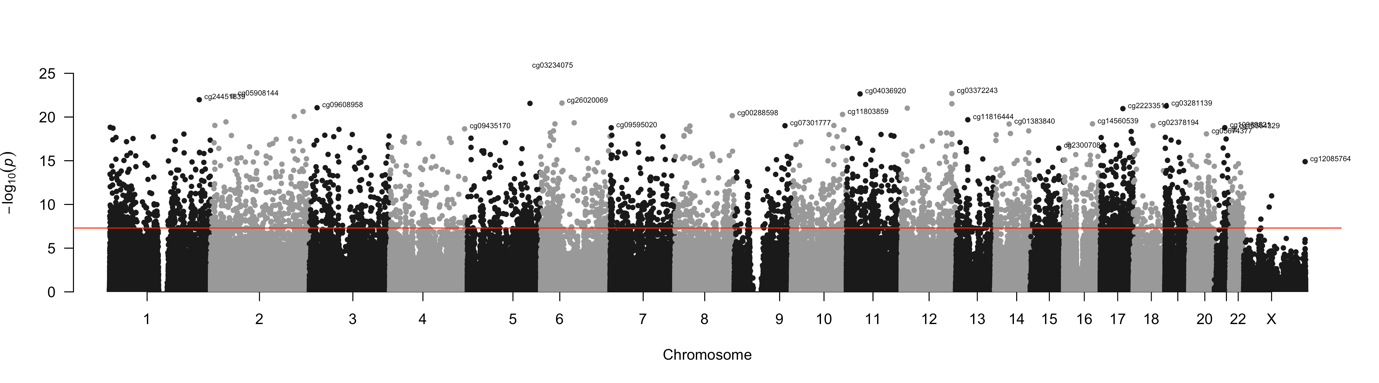


**Additional file – Figure 11**. Manhattan plot presenting association results between chemotherapy (samples obtained 0.5 to 2 years from chemotherapy start date vs. no chemotherapy) and 525,100 DNA methylation in CpG probes in breast cancer patients with saliva specimens. Presented here are associations that reached statistical significance at a Bonferroni threshold p-value (p<9.52e-8) with adjustments for batch, immune cell content (LUMP), age at breast cancer diagnosis, and ethnicity (Additional file 13).


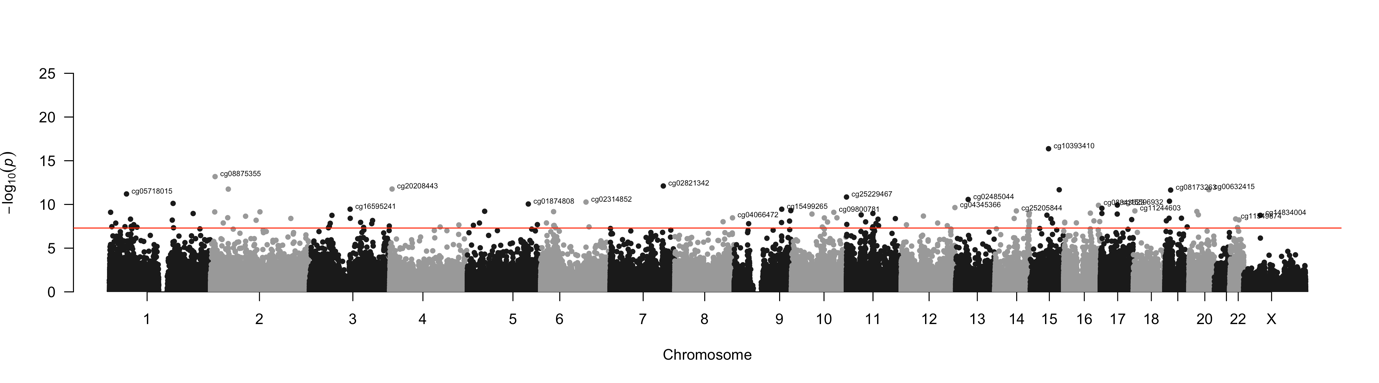


**Additional file – Figure 12**. Manhattan plot presenting association results between chemotherapy (Samples obtained >2 years from chemotherapy start date vs. no chemotherapy) and 525,100 DNA methylation in CpG probes in breast cancer patients with saliva specimens. Presented here are associations that reached statistical significance at a Bonferroni threshold p-value (p<9.52e-8) with adjustments for batch, immune cell content (LUMP), age at breast cancer diagnosis, and ethnicity (Additional file 13).


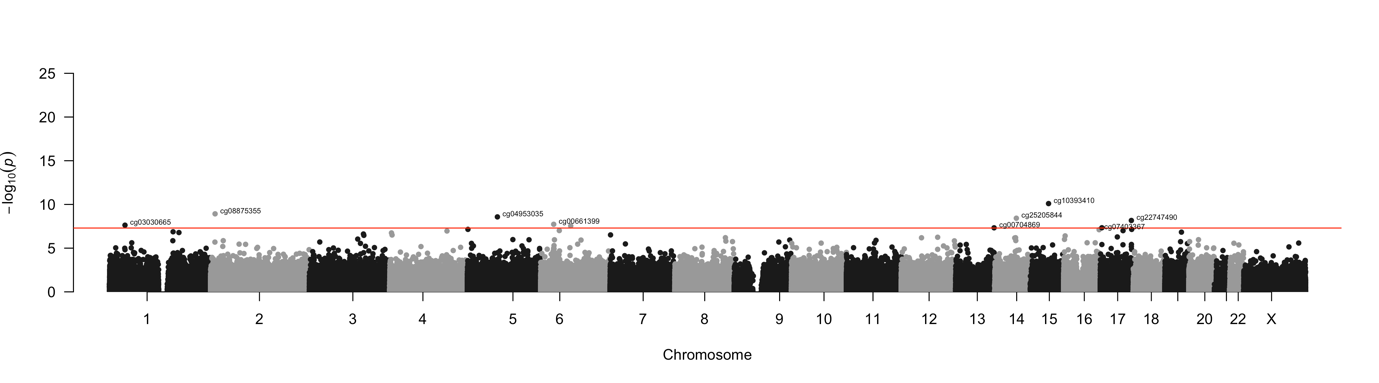

Supplement: Supplementary file 8 — Additional file 8. Additional file - Figures 1 to 12. [file 13058_2023_1730_MOESM8_ESM.docx]
